# Harmful effects of lithium-ion battery thermal runaway: scale-up tests from cell to second-life modules

**DOI:** 10.1039/d3ra02881j

**Published:** 2023-07-10

**Authors:** Rico Tschirschwitz, Christopher Bernardy, Patrick Wagner, Tim Rappsilber, Christian Liebner, Sarah-K. Hahn, Ulrich Krause

**Affiliations:** a Bundesanstalt für Materialforschung und -prüfung (BAM) Unter den Eichen 87 12205 Berlin Germany rico.tschirschwitz@bam.de; b German Fire Protection Association (Vereinigung zur Förderung des Deutschen Brandschutzes e.V.—vfdb) Wolbecker Straße 237 48155 Münster Germany rico.tschirschwitz@bam.de; c Faculty of Process- and Systems Engineering, Institute of Apparatus and Environmental Technology, Otto von Guericke University of Magdeburg Universitätsplatz 2 39106 Magdeburg Germany

## Abstract

For a comprehensive safety assessment of stationary lithium-ion-battery applications, it is necessary to better understand the consequences of thermal runaway (TR). In this study, experimental tests comprising twelve TR experiments including four single-cell tests, two cell stack tests and six second-life module tests (2.65 kW h and 6.85 kW h) with an NMC-cathode under similar initial conditions were conducted. The temperature (direct at cells/modules and in near field), mass loss, cell/module voltage, and qualitative vent gas composition (Fourier transform infrared (FTIR) and diode laser spectroscopy (DLS) for HF) were measured. The results of the tests showed that the battery TR is accompanied by severe and in some cases violent chemical reactions. In most cases, TR was not accompanied by pre-gassing of the modules. Jet flames up to a length of 5 m and fragment throwing to distances to more than 30 m were detected. The TR of the tested modules was accompanied by significant mass loss of up to 82%. The maximum HF concentration measured was 76 ppm, whereby the measured HF concentrations in the module tests were not necessarily higher than that in the cell stack tests. Subsequently, an explosion of the released vent gas occurred in one of the tests, resulting in the intensification of the negative consequences. According to the evaluation of the gas measurements with regard to toxicity base on the “Acute Exposure Guideline Levels” (AEGL), there is some concern with regards to CO, which may be equally as important to consider as the release of HF.

## Abstract 2

Unique scaled up thermal runaway tests were performed with lithium-ion batteries from the cell level to module level for stationary applications.

## Graphical abstract

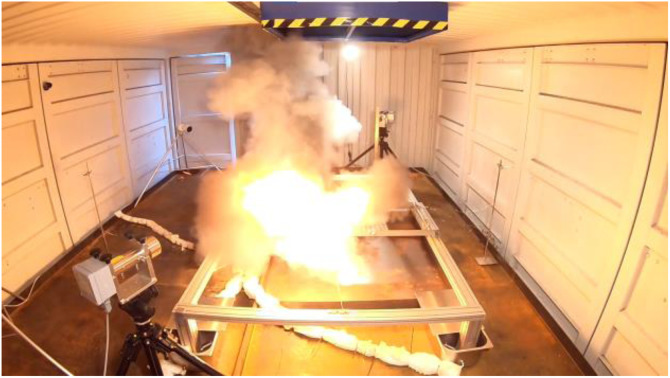

## 1. Introduction

With the increasing use of renewable sources for the production of electricity, energy storage systems are becoming an essential component for the stabilization of the power grid. In this case, stationary battery storages have been proven to be a solution to the intermittent nature of renewable sources, which is a significant issue. Globally, the installed battery power capacity has increased over the last few years,^1^ with a further increase expected, especially in the USA^2^ and Europe.^3^ Stationary energy storage system applications are relevant to power stations, industrial consumers, and residential buildings, optimizing the time-of-use/energy cost^4^ in the case of own energy co-production. Further, the use of second-life batteries for stationary applications after the end of their first intended life, *e.g.*, in the field of e-mobility,^5^ is becoming increasingly relevant in the above-mentioned applications.

One of the main challenges associated with the use of lithium-ion batteries is managing the potential risk of thermal runaway (TR). This hazardous event can occur in all types of lithium-ion batteries, ranging from a single cell^6–9^ to installed grid-scale storage applications.^10–12^ The sequence of events leading to the occurrence of TR has been described in the literature.^13–18^ TR in batteries can result in the release of a large amount of heat^19–21^ and gas, which can be toxic and flammable based on its composition.^22,23^ In the final stage of TR, the battery may even produce flames and explode.^24,25^ Alternatively, it can result in the release of a large amount of flammable gases, which generates an ignitable atmosphere, subsequently resulting in a gas explosion.^26^ It must be considered that even TR in a single cell can initiate its propagation to neighboring cells or larger units such as modules and entire batteries.^27–30^

It is well known that safety is one of the key factors for the social acceptance of technology. Thus, the fear of TR in batteries or even an explosion hinders the development and commercialization of this particular new technology. In explosion protection, the safety concept classification of primary, secondary, and tertiary explosion protection has been established. Specifically, primary explosion protection includes all measures that prevent the generation of an explosive atmosphere, whereas secondary explosion protection involves all activities that avoid the presence of an ignition source. In the case of tertiary explosion protection, measures are adopted to minimize the effects of unavoidable explosions.^31^ Adapting this explosion protection approach in the TR of batteries, the primary and secondary measures prevent TR. In this field, manifold solutions and research approaches have been pursued.^32,33^ The typical strategies in this context are the early phase detection of critical conditions,^34–38^ use of other/new cell chemistry with lower or no risk of TR,^39,40^ and composition of batteries with different cell chemistries to change the environmental conditions towards reducing the risk of TR.^41^ Another promising approach is to optimize the battery design, either by minimizing the heat exchange between different sections^42,43^ or optimizing the cooling conditions.^44^

All these risk-minimization strategies can reduce the probability of a propagating TR, but not rule it out completely. For example, an approach such as the use of new or safer cell chemistries is not feasible for the utilization of second-life batteries in stationary applications. At present, the batteries in question are already in use. In this case, battery state analysis, followed by a risk assessment to address the consequences of TR of entire stationary battery applications is necessary. Subsequently, the determined parameters should be used to set up appropriate safety precautions (*e.g.*, in the areas of constructional fire protection, process and plant safety and firefighting). Increasing the safety level also increases the confidence of the society in this technology.

Finally, given that TR cannot be ruled out completely, tertiary explosion protection measures must be considered. As input parameters, information about the release of gases^22,23^ and heat (the thermal impact on the environment)^19–21,45,46^ is required. Experimental data for the heat release rate (HRR) and the total heat release (THR) have been reported in the recent literature. All types of cell geometry (*i.e.*, cylindrical,^9,47–58^ pouch,^20,59–64^ and prismatic^60,65–67^) and cathode materials (NMC,^25,48–50,55,58,61,63–66,68^ LFP,^9,20,48,49,51,56–61,65^ NCA,^47,60,69^ LCO,^20,47,48,51–54,58,60,65,67,69^ and LMO^47,62,65,68^) are considered. However, most safety-related research data is available for cells or smaller storage up to a capacity of *C* ≤ 35 A h.^9,20,25,47–57,59–62,65–69^ This only allows a limited statement regarding the THR from whole stationary storage or (only) parts of it.

Similarly, most gas release data have been published for smaller cells. This applies to both the total amount of released gas^23,70–77^ and qualitative analysis of the gas components^23,51,62,74,78–83^ (with special focus on hydrogen fluoride (HF)^20,59,60,62,74,79,84^).

Some experimental work involving TR experiments with occasionally so-called “large-scale” or “large-format” batteries involved cell experiments (one or more) with a capacity in the range of *C* = 100 A h^19,60,85–101^ to *C* = 200 A h.^29,102–105^ Some large-/real-scale tests were carried out to examine the burning behavior of stored cells in a warehouse. It must be considered that for these tests, a large number of single pouch cells^106^ and cylindrical 18 650 cells^107^ were used. In similar tests, the U.S. Department of Transportation analyzed the vent gas of 18 650 cells. More than 500 cells were used per test in a chamber with *V* = 10.8 m^3^.^108^ However, the results of these tests with a large quantity of single cells only allows limited comparability regarding the burning behavior and consequences of TR in stationary storage modules. Based on the energy content, a single module for stationary applications can correspond to several hundred cells with the size of 18 650. In the case of TR, it can result in several hundred “small” TRs over a longer time span or one big TR. Several other test series or single tests deal with the extinguishment of battery fires,^106,109–112^ and the subsequent exposure to hazardous substances.^113^ Tests with fire suppression allow no statement about the worst-case consequences due to the fact that the TR reaction is diminished, suppressed or interrupted. A series of four full-scale vehicle fire tests, two electrical vehicle (EV) and two combustion engine vehicles was performed by Lecocq *et al.*^114^ Further tests in this field were carried out by Willstrand *et al.*^115^ However, considering the assessment of battery TR consequences, this study was limited because entire cars were tested.^114,115^ Thus, the fire load was not only driven by the battery, the battery itself was not placed in a representative confined space (impact on consequences).

Only few large/real-scale (*C* > 200 A h) experimental studies on the consequences of battery TR has been reported. Liu *et al.* analyzed the TR behavior, the released gas and the HRR/THR of LFP/graphite-batteries made of two cells with a capacity of *C* = 243 A h.^116^ Mao *et al.* conducted tests with 300 A h LFP-batteries to investigate the different phases of TR, the gas and heat release and the impact of the state of charge (SOC).^117^ The results for a single experiment with a 12 cell module (*E* = 1.65 kW h) with NMC/graphite-electrodes were reported by Cheng *et al.* One of the key aspects of this test was the TR propagation from cell-to-cell inside the module.^118^ Gao *et al.* analyzed both the propagation from cell-to-cell and module-to-module in an EV battery pack (8 modules with 12 cells each) with a stored electrical energy of *E* = 13 kW h.^30^

Two full-scale fire tests with powerpacks (16 energy storage pods, 900 cells per pod, type 18 650-cells) were performed by the Fire Protection Research Foundation on behalf of the NFPA. One test was initiated inside using a cartridge heater, whereas in the second test, an external propane burner was applied. The key findings were an HF concentration in the test with the external heating of *c*_HF_ > 100 ppm, and in the other test, a concentration of *c*_HF_ = 26 ppm. No explosions or projectiles were observed during these tests.^119^

To date, the comprehensive safety assessment of lithium-ion-batteries is very sophisticated due to the limited amount of relevant data available, as explained above. Thus, the experimental investigations described herein intend to fill this gap. Based on the evaluation of the experimental results, the data generated is more applicable for process and plant safety assessment, constructional fire protection, and firefighting. To obtain more information on the scalability of the cell-test results, the experimental program was comprised of both cell tests (single and stacks) and module tests (two different sizes) under comparable conditions.

## 2. Experimental

### 2.1 Test program and experimental setup

Twelve full-scale fire tests were conducted in total, as listed in Table 1. All tests were performed under almost comparable initial conditions. Accordingly, it should be possible to compare the cell experiments with the module tests. All tests were carried out in a large-scale test stand for real-scale fire tests of stationary electrical storage systems.

**Table 1:** Experimental program

Test no.	Unit tested			TR initiation method	*T*_ambient_ (°C) initial test temperature
	Cell	Three-cell-stack	Module		
#01	X	—	—	Overheating	31
#02	X	—	—	Overheating	24
#03	X	—	—	Overheating	24
#04	X	—	—	Overheating	25
#05	—	X	—	Overheating	21
#06	—	X	—	Overheating	21
#07	—	—	X	Overheating	19
#08	—	—	X	Overheating	21
#09	—	—	X	Overheating	30
#10	—	—	X	Overheating	25
#11	—	—	X	Overloading	22
#12	—	—	X	Overheating	20

**Fig. 1:**
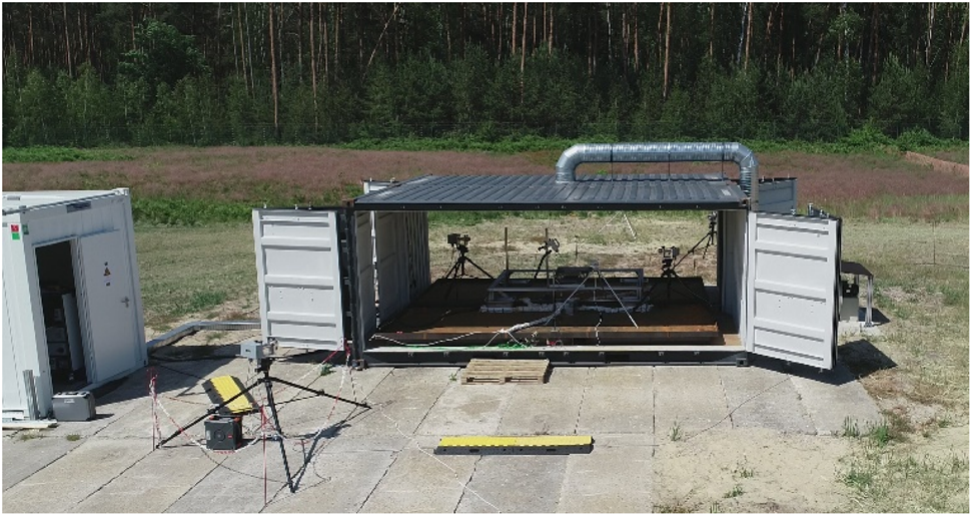
Test stand in the all-side open configuration.

The main part of the test stand is depicted in Fig. 1. This part consisted of two 20′-side-door-containers. The back walls of the two containers were removed and the two containers were joined facing each other with their sides open. This experimental room could be opened from each side individually or closed completely. In the centre of the roof, a ventilation system was installed to exhaust the smoke gases after the tests with the closed-side configuration. A 10′-office-container was used for the data acquisition systems and the Fourier-transform infrared spectroscopy (FTIR) analyzer was placed next to the experimental room (Fig. 1: left side, white container). These two parts of the test stand were positioned in an open field, and consequently a safety zone with a radius of *r* = 100 m could be easily established during the test. The remote-control room for regulating and monitoring the experiments was located outside the safety zone. Six IP-cameras inside and outside the experimental room were used to monitor the test sample and safety zone.

### 2.2 Gas measurement technique

**Fig. 2:**
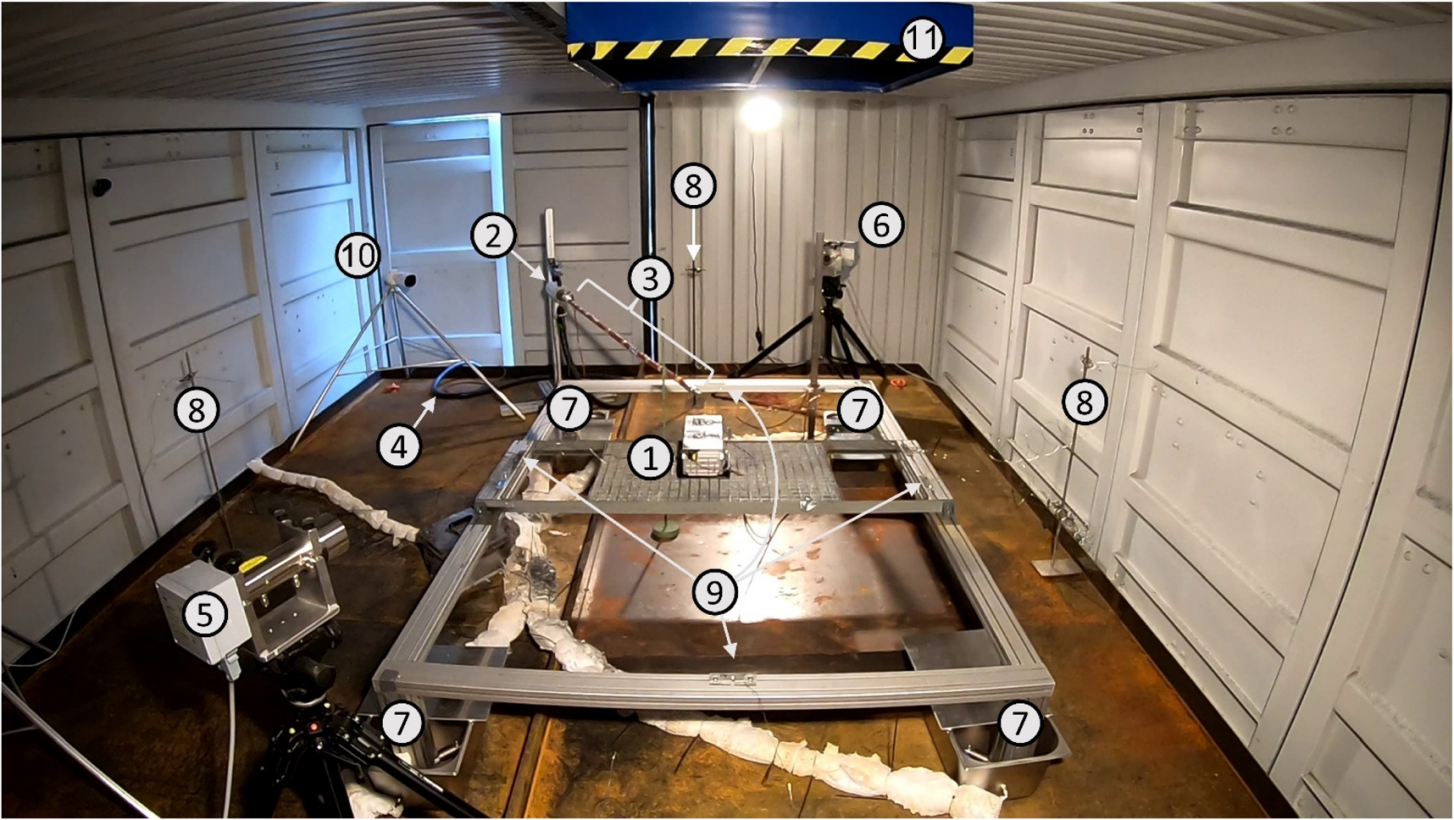
Measurement setup (test #07): Sample desk with module (*E* = 2.65 kW h) (1), heatable gas sampling probe with pre-filter for FTIR (2), heatable sampling lance for FTIR sampling probe, *l* = 2 m (3), heatable sample gas line (4), HF diode laser spectrometer, receiver (5) and transmitter (6), load cells at each table leg of the sample desk, positioned in a cooling container (7), type K thermocouple, distance to test sample *l* = 2 m (8) and *l* = 1 m (9), IP-camera (10), and exhaust hood (11).

Two different gas measurement devices were used. The first one was an FTIR gas analyzer designed for hot combustion products and the second a diode laser spectrometer (DLS) to measure the average value of the HF concentration (averaged over the length of the analytical laser beam). Both devices were partially visible in the experimental room (Fig. 2: no. 2–4 FTIR, 5–6 DLS).

FTIR spectroscopy was performed using a Gasmet CX4000 FTIR with the Calcmet 7 software (Gasmet Technologies GmbH, Germany), which was suitable for multi-gas analysis. An optical length of *l* = 5.00 m and wavenumber range of *ṽ* = (900–4200) cm^−1^ at a resolution of *ṽ* = 7.7 cm^−1^ were employed. Herein, a reported single measurement point was derived by averaging 50 spectra, *i.e.*, the FTIR spectrometer recorded 10 spectra per second, which was set to a measuring time of Δ*t* = 5 s. The internal heating was set to *T* = 180 °C to prevent the sample gas from condensing on the inner walls of the measuring cell, tubes, fittings, filters, and sample tube. Therefore, a hot gas conditioning system (SYCOS P-HOT-QL17, Gasmet Technologies GmbH, Germany) was used for gas sampling. A dilution system diluted the gases by a factor of about 1/35 with nitrogen before they were transported to the measuring cell. The dilution factor was calculated for each measurement point corresponding to the dilution gas flow. The residual gas from the FTIR was pumped into a condensation trap, and subsequently into a paramagnetic oxygen analyzer (PMA30, M&C, Germany).

Both the sampling system and the two analyzers (FTIR and oxygen) were positioned in the measurement container (*cf.*: Fig. 1). From there, a heatable sample gas line (*l* = 10 m) was connected to the experimental room (Fig. 2: no. 4). After the sample gas line, a gas sampling probe with pre-filter (Fig. 2: no. 2) was mounted on a tripod at a distance of *l* = 2 m from the battery to be tested. To protect the sampling probe and filter from being damaged, a heatable sampling lance was used to transport the gas sample to the FTIR spectrometer directly above the cell(s)/module (Fig. 2: no. 3; distance sample – suction point lance: *l* = 0.2 m). Calibration of the FTIR spectrometer was performed by the manufacturer for the substances listed in Table 2 in the corresponding measuring range. The dilution unit enlarged the measuring ranges proportional to the dilution factor.

**Table 2:** FTIR, substances calibrated with corresponding measuring range

Substance	Measuring range [ppm]
CO_2_	0–300 000
H_2_O	0–100 000
CO	0–10 000
CH_4_, ethanol	0–2000
C_2_H_6_, C_3_H_8_, HCl, ethylene carbonate (EC)	0–1000
Diethyl carbonate (DEC), methanol, cyclohexane, formaldehyde, C_3_H_8_, acetaldehyde	0–500
C_3_H_6_	0–200
C_2_H_4_, HF, ethyl methyl carbonate (EMC), POF_3_, propylene carbonate, N_2_O, acetylene	0–100
Dimethyl carbonate (DMC)	0–20

The DLS (LaserGas™ II Monitor, NEO Monitors AS, Norway) for HF worked based on single-line spectroscopy in the near infrared range (*λ* = 1322.5 nm). The measuring range depended on the transmitter-receiver-distance. In the configuration with a distance of *l* = 10 m, the measuring range was *c*_HF_ = (0–200) ppm, whereas a reduced distance of *l* = 1 m resulted in a measuring range of *c*_HF_ = (0–2000) ppm. Optical measurement systems require a sufficient transmission. However, the gases and smoke released during the tests may reduce the transmission between the transmitter and receiver. Thus, the employed DLS allowed measurements with no influence on the standard deviation up to a transmission of *τ* ≥ 5%. A measurement with a lower transmission *τ* = (5–1)% was still possible but it increased the measurement uncertainty. The transmitter (Fig. 2: no. 6) and receiver (Fig. 2: no. 5) were positioned at a distance of *l* = 3.9 m. The line measurement was carried out at a height of *l* = 0.5 m above the test sample. The system was protected against surrounding impacts according ingress protection code IP 66. A separate program (GAS MONITOR LG II/LG Q, NEO Monitors AS, Norway) was used for data acquisition.

The FTIR and DLS were used in tests #1–#9. Additionally, the DLS was used in test #12. Given that powerful reactions were expected due to the size of the test samples, the gas measurement inside the experimental room was dispensed in tests #10 and #11. A second DLS was used in tests #10 and #12 for the measurement at the end of the exhaust duct.

### 2.3 Additional measurement technique

Two modular data loggers (Keysight DAQ970A, Keysight Technologies, US) were used for data acquisition. Both data loggers were equipped with three 16-channel-multiplexer-modules (Keysight DAQM902A, Keysight Technologies, US) each. The Keysight BenchVue DAQ software was used for data visualization and test monitoring. The parameters recorded were the temperature, load cell signal, and sample voltage. One cell/module voltage channel was used in all the tests, except in tests #5 and #6, where three voltage signals were recorded (Table 1). The acquisition frequency for the voltage was chosen with *f* = 1 Hz.

Eight temperature sensors were used to measure the temperature inside the experimental room, *i.e.*, four at a distance of *l* = 2 m (*h* = 1 m; Fig. 2: no. 8) and another four with *l* = 1 m (*h* = 0.5 m; Fig. 2: no. 9) from the tested cell(s)/module. For the temperature measurement of the test samples, either four sensors in the single-cell tests (Table 1: #1–#4), eight sensors in the cell stack tests (Table 1: #5 and #6) or ten sensors in the module tests (Table 1: #7–#12) were installed. For all 18 temperature channels in maximum, sheathed thermocouples type K, class 1 according to IEC 60584-1,^120^ with a diameter *d* = 1.5 mm were used (deviation in maximum Δ*T* = ± 1.5 K or Δ*T* = 0.004 × *T* [°C]). The measurement frequency was also *f* = 1 Hz on all channels. Signal amplification was performed using a J.E.T. amplifier (J.E.T. Type Bedo SAM 146, J.E.T. Systemtechnik, Germany).

The test samples were placed on an iron sample desk. Each of the four table legs of the sample desk was equipped with a bending beam-type load cell (SHBxR, Revere Transducers Europe, Vishay Precision Group, UK). Each load cell provided a measuring range of *m* = (0–350) kg. Being protected against the ingress of water and dust according code IP 68, stainless steel buckets filled with water (Fig. 2: no. 7) were used to protect the load cells against high temperature from the battery fire. The load cells were used in tests #7–#12 (Table 1).

A camera system (Bascom, Germany), consisting of a recorder (R8XK) and six cameras (one bullet camera PB40K (Fig. 2: no. 10) and two XD10K-A dome cameras inside, three PB40K bullet cameras outside, all cameras IP 66), was used for the experimental documentation and monitoring of the tests. The recording frequency was *f* = 25 fps. Additionally, two further cameras (GoPro Hero 9, GoPro, US) were used in selected tests (*f* = 60 fps). In the tests where the fragments of the samples were ejected, the throwing distances were measured manually with a measuring tape after the test.

### 2.4 Battery cells/modules and test conditions

Table 3 lists the specifications of the tested cells and modules. The active material of the cathode was NMC for all the test samples. The cells in tests number #1–#6 were from the same manufacturer and batch. The three small modules (Table 3: #7–#9) and the three large modules (Table 3: #10–#12) had the same number of cells, nominal stored electrical energy, weight, and dimensions. Each one of the three modules had a higher voltage than the other two because of the different cell configuration and internal wiring (Table 3: #9 and #12). All the modules came from battery electric vehicles, were used and at the stage where they could be used for their second life in stationary applications. All the cells/modules used for the experiments were previously charged to a state of charge of (nearly) SOC = 100%. The only exception was the module of test #11 with an SOC of ≫100%. Here, the initiation method overload was applied. Due the general setup of the test stand on an open-air test site (Fig. 1), the tests were performed under ambient conditions on a specific day. As can be seen in Table 1, the ambient temperature at the beginning of the tests was in the range of *T* = (19–31) °C.

**Table 3:** Specifications of the test samples

Test no.	No. of cells	Configuration	Voltage [V]	Nominal electrical energy stored [kW h]	Mass [kg]	Dimension [cm]
#01–#04	1	—	4.16–4.18	0.0525	0.26	19 × 8.8 × 0.76
#05–#06	3	—	All 4.19	0.1575	0.78	19 × 8.8 × 2.28
#07	12 (in 1 module)	3S4P	12.16	2.65	13.0	40 × 16 × 10
#08	12 (in 1 module)	3S4P	12.26	2.65	13.2	40 × 16 × 10
#09	12 (in 1 module)	6S2P	25.40	2.65	12.7	40 × 16 × 10
#10	24 (in 1 module)	8S3P	31.00	6.85	30.8	59 × 22.5 × 11
#11	24 (in 1 module)	8S3P	33.00	6.85	30.9	59 × 22.5 × 11
#12	24 (in 1 module)	12S2P	47.59	6.85	31.1	59 × 22.5 × 11

### 2.5 Test procedure

After the charging process, the cells/modules to be tested were placed on the sample desk (Fig. 2: no. 1) on a standard single heating plate with a diameter of *d* = 18 cm. For the single cell tests (Table 1: #01–#04), a second heating plate was used reverse from the top, given that the pre-tests showed that the heat dissipation was very large when using a heating plate from below alone, and consequently the single cells could not go into TR with the selected initiation method. In the case of the modules with a length of *l* = 59 cm (Table 3), two heating plates were used (Table 1: #10 and #12). At the beginning of the experiments (Table 3: #1–#6), the cells were positioned on a pre-stressed aluminum plate (250 × 400 × 3 mm). During tests #7–#12, the modules were placed directly on the heating plate. From test #8, the modules were fixed and fastened on the sample desk with threaded rods to prevent their displacement during TR. Between each heating plate and cell/module, a thermocouple was placed in a milled slot of the heating plate to reduce the influence on its heat transfer. The heating plates were manually controlled using an isolating variable ratio transformer to keep the surface temperature of the heating plate in the range of *T* = (370–420) °C. The advantage of using a laboratory isolating transformer was that the heating plates were not pulsed. By varying the voltage, the current and the desired heating power at the plates could be adjusted. Due to the large safety distance, a larger cross-sectional area was used in the supply line of the heating plates to minimize the line losses (250 m H07RN-F 5 G 6.0 mm^2^). The used heat supply ensured a homogeneous and continuous heat input in the test samples, as already described in the literature.^96^ The energy introduced *via* the isolating transformer was measured and documented using an energy meter. For the overcharging test (Table 1: #11), two series-connected laboratory power supplies (Voltcraft type PPS-16005, Conrad Electronic SE, Germany) were used for charging. The electrical charging current was *I* = 10 A.

As described in Section 2.1, all sides of the test stand were openable. On the one hand, it must be considered that open doors guaranteed good visibility during the tests, but the gas measurement was also strongly influenced by natural convection. On the other hand, completely locked doors could result in significant damage if an ignitable atmosphere is formed due to the gas release during the TR (Section 1), where a subsequent gas explosion in the locked experimental room would develop high explosion pressures because of the confinement. Therefor one door was not locked but left ajar (test #1–#9: Fig. 1, left door, test #10–#12: Fig. 1, front door). In case of a gas explosion, this door worked as a pressure relief and diminished the explosion pressure. With only one door ajar, the experimental room was fully closed. Accordingly, FTIR gas measurement less influenced by the external flow was possible, as shown in comparative measurements in the fire tests in closed rooms and in the free field.^121^

The starting time, *t*_0_, of a single test run was defined as the moment when the heating was switched on and overcharging (switch on laboratory power supply) initiated.

## 3. Results and discussion

### 3.1 Initiation and conditions of cells/modules during TR

In this section, we present the conditions before TR occurred (temperature and heat input) and at the moment of TR (time to TR, temperature, and voltage). Furthermore, the damaging event and its mechanism leading to the destruction of the cells/modules and the mass burn-up are discussed.

#### 3.1.1 Definition of the TR condition

**Fig. 3:**
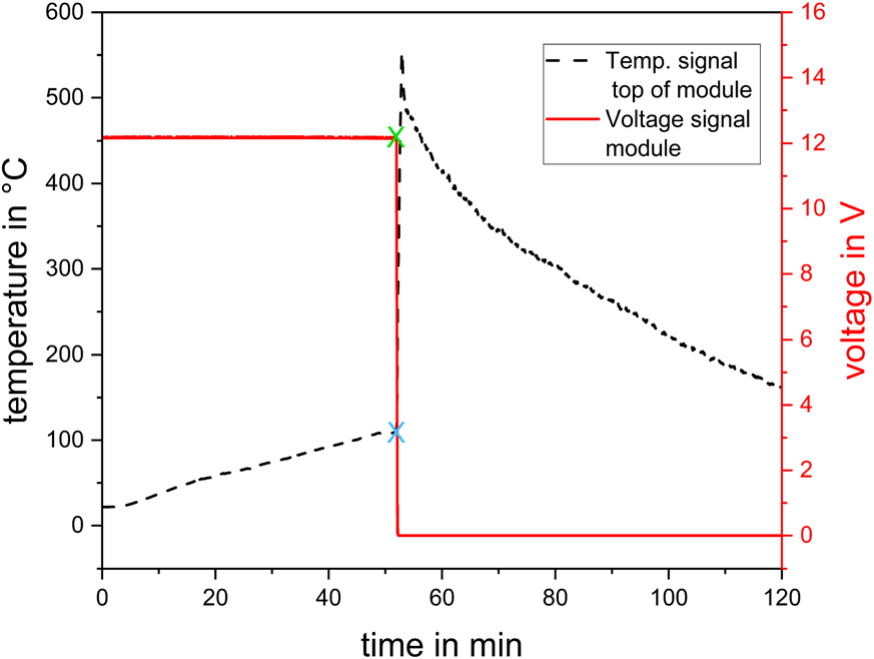
Test #07, temperature central top of module and module voltage, green cross: starting point for the sudden voltage drop (Δ*t*_*U* drop_) and blue cross: starting point for the sudden temperature increase (*T*_onset_, Δ*t*_*T*_onset__).

During the heating and overcharging experiments, all the samples underwent TR. In the all tests where the TR was initialized by a heating plate, the temperature measured on the central top of the cell/module revealed a characteristic trend. Given that the heat flow was conducted from the bottom to the top side (except in test #01–#04, where a second heating plate was placed on top of the cell (Section 2.5)), there was no section of the test object that could assumed to be at a lower temperature. Consequently, this is the temperature signal used for evaluating the TR progress. A typical temperature and voltage signal (test #07) is depicted in Fig. 3.

Heating was initiated at *t*_0_ = 0 min. The temperature of the heating plate increased and the test sample followed corresponding its thermal inertia. After a short delay time, the increase in temperature was approximately linear. The input parameters of the heating process, *i.e.*, heating power and heating energy, are listed in Table 4.

**Table 4:** Initiation of TR by heating: heating power and heating energy per test

Test no.	Heating power [W]	Heating energy [kW h]
#01	500	0.090
#02	500	0.088
#03	660	0.100
#04	666	0.111
#05	600	0.150
#06	519	0.150
#07	968	0.823
#08	704	0.865
#09	714	0.660
#10	1368	1.145
#11	—	—
#12	1379	1.770

Due to the heat generated by the chemical reactions leading to TR, a sudden increase in temperature was detected. The transition from the heating phase to the TR reaction is marked with a blue cross in Fig. 3, which refers to *t*_*T*_onset__ and *T*_onset_. The time span from *t*_0_ to *t*_*T*_onset__ is denoted as Δ*t*_*T*_onset__. A few seconds before or after *t*_*T*_onset__, a sudden voltage drop was detected, which is marked with a green cross in Fig. 3, referring to *t*_*U* drop_, indicating the decomposition of the separator and collapse of the battery.^13–18^ The time span from *t*_0_ to the beginning of the voltage drop *t*_*U* drop_ is denoted as Δ*t*_*U* drop_.

The biunique indicator for TR is the combination of this sudden increase in temperature at the top surface of the cell/module combined with the sudden drop in the measured voltage of the cell/module. The time at which both criteria are met, *i.e.*, when TR occurs, is denoted as *t*_TR_, whereas the duration from the starting time *t*_0_ to TR (*t*_TR_ − *t*_0_) is denoted as Δ*t*_TR_. Table 5 presents Δ*t*_*T*_onset__, Δ*t*_*U* drop_, Δ*t*_TR_ and the start temperature of TR, *T*_onset_.

**Table 5:** Time to voltage drop (Δ*t*_*U* drop_, criterion 1), onset temperature on sample top after heating (*T*_onset_) and time to onset temperature (Δ*t*_*T*_onset__, criterion 2), as defined in Fig. 3, time gap between both (Δ*t*_*T*_onset__ − Δ*t*_*U* drop_) and time to TR (Δ*t*_TR_), when both criteria are fulfilled

Test no.	Δ*t*_*U* drop_ [s]	Δ*t*_*T*_onset__ [s]	*T*_onset_ [°C]	Δ*t*_*T*_onset__ − Δ*t*_*U* drop_ [s]	Δ*t*_TR_ [min : s]
#01	726	729	303	3	12 : 09
#02	644	648	282	4	10 : 48
#03	636	640	298	4	10 : 40
#04	637	640	294	3	10 : 40
#05	933	943	86	10	15 : 43
#06	1114	1103	90	−11	18 : 23
#07	3117	3116	109	−1	51 : 57
#08	4393	4430	119	37	73 : 50
#09	3409	3409	135	0	56 : 49
#10	2964	2976	123	12	49 : 36
#11	—	23 880	118	—	398 : 00
#12	4617	4624	138	7	77 : 04

In the overcharging test, #11, the voltage signal was lost shortly after the beginning of the test (the alligator clip on one pole slipped off). Thus, only Δ*t*_*T*_onset__ is given. In tests #05 and #06, *i.e.*, the cell stacks, the signal of the temperature sensor at the top of the lowest cell (between cells 1 and 2) was used for the determination of Δ*t*_*T*_onset__ and *T*_onset_. After reaching the maximum, the temperature decreased exponentially to the ambient conditions (Fig. 3).

#### 3.1.2 Cell experiments

The time to TR in the single cell test was in the range of Δ*t*_TR_ = (640–729) s. This time depends on the initiation method and heating power. For example, the results reported by Wang *et al.*^92^ show nearly the same tendency of heating time. In these experiments, NMC prismatic cells (*C* = 50 A h) were used and heated by an electric furnace with an electrical power of *P* = 400 W. The resulting times to TR were about Δ*t*_TR_ = 750 s. In contrast, the overheating tests of NMC pouch cells (*C* = 14 A h) reported by Sturk *et al.*^61^ showed a time to TR of approximately Δ*t*_TR_ = 30 s. The burner used for this purpose, according to the single burning item test standard (EN13823^122^), provided a thermal power of *Q̇* = 15 kW, which is 22–30 times the values presented in Table 4 (#1–#4). Thus, these different Δ*t*_TR_ values do not conflict with each other. The presently reported onset temperature for TR in the single cell experiment is in the range of *T*_onset_ = (282–303) °C (Table 5). Similar tests by other researchers, such as Koch *et al.* (*T*_onset_ = (150–250) °C)^90^ and Amano *et al.* (*T*_onset_ = (165–189) °C),^77^ measured lower onset temperatures. The reason for the significantly higher *T*_onset_ in the tests reported herein is that the second heating plate increased the heating surface, and thus reduced the heat losses, while in certain cases, TR could not be initiated with a single heating plate (Section 2.5). This is also reflected in *T*_onset_ for the cell stack experiments (Table 5, #05 and #06). In these tests, only one heating plate was used from below. Consequently, TR started at temperatures of *T*_onset_ = (86 and 90) °C. A second result of using only one single heating plate was the slightly higher time to TR (Table 5, #5 and #6: Δ*t*_TR_ = (943 and 1103) s, respectively).

**Fig. 4:**
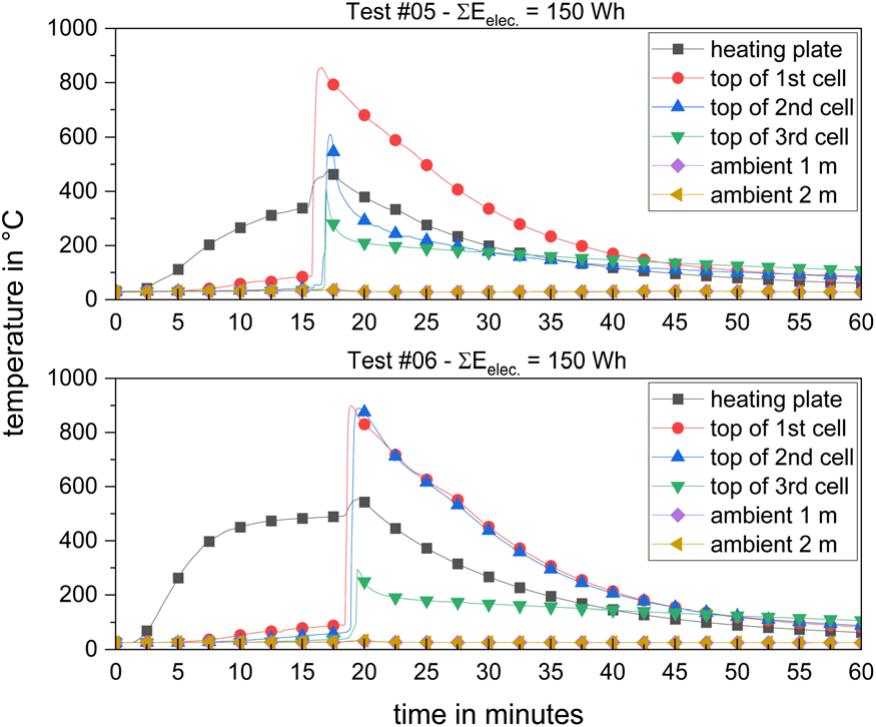
Temperature–time–histories of TR experiments with 3-cell-stacks, temperature of the heating plate (below 1st cell) and temperature of the top of 1st, 2nd and 3rd cell and ambient temperatures at distances of *d* = (1; 2) m.

**Fig. 5:**
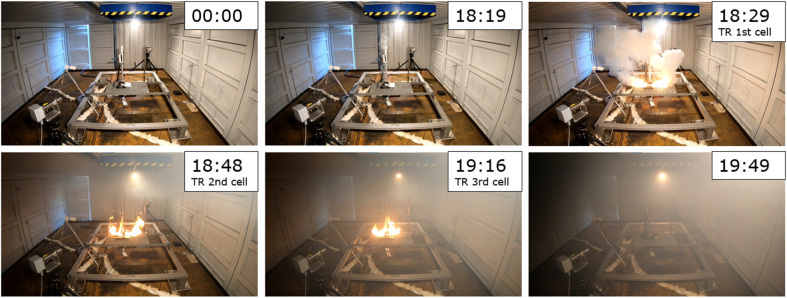
Progress of three-cell stack test (test #06, time after start of the test).

In the single cell tests, the maximum temperature measured at the cell top was in the range of *T*_max_ = (470–515) °C. In the cell stack tests, the maximum temperature was significantly higher, *i.e.*, *T*_max_ = (854 and 899) °C. The temperature–time–histories of tests #05 and #06 over the entire stacks are depicted in Fig. 4. The bottom cell (1st) went into TR first, followed by the neighbouring cell above (2nd) and the top cell followed at last (3rd). Fast propagation behaviour, *i.e.*, small follow-on times from the 1st to 3rd cell TR was obtained in both experiments. The successive propagation progress is visible in the video recordings. Fig. 5 shows a sequence of pictures taken from the video. Slight degassing started about Δ*t* = 18 min after the start of the test. Another couple of seconds later (Δ*t* = 10 s), outgassing intensified significantly, while the first cell underwent TR. Additionally, a jet flame with a length of *l* = 50 cm at both ends of the cell was visible (Fig. 5: top, right). Another Δ*t* = 20 s later, the 2nd cell reached *T*_onset_. The TR of the 3rd cell started an additional Δ*t* = 40 s after the reaction of the 2nd cell. Each cell TR was accompanied by one jet flame. The total gas release after all three cells underwent TR filled the experimental room (*V* = 75 m^3^) with dense smoke (Fig. 5: bottom, left). Approximately Δ*t* = 60 min after the start of test, the temperature decreased to *T* = 80 °C between the cell and heating plate. The progress of the other cell-stack test (#05) supports the findings from test #06. The relative mass loss of the cell stacks was Δ*m*/*m*_0_ ≈ 53% in test #05 and Δ*m*/*m*_0_ ≈ 27% in test #06. This variation in mass loss was previously reported in TR experiments, *e.g.*, Koch *et al.* Δ*m*/*m*_0_ ≈ (20–55)% for NMC pouch cells,^90^ and Chen *et al.* reported Δ*m*/*m*_0_ ≈ (29.0–54.8)% for cylindrical LFP cells with 100% SOC.^56^

#### 3.1.3 Small module tests

**Fig. 6:**
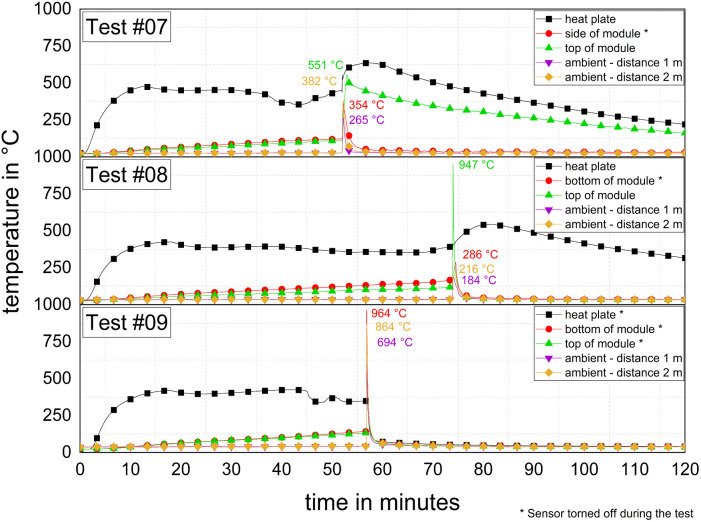
TR experiments with 2.65 kW h modules (test #07–#09), temperature of the heating plate, top of the module, side respectively, bottom of module and in ambient conditions at distances of *l* = (1; 2) m.

Fig. 6 depicts the temperature–time–histories of the 2.65-kWh-tests (Table 1: #07–#09). Within the first minutes, the temperature curves of the heating plates showed an almost linear increase, similar to the cell tests before. After Δ*t* = 15 min, the temperature reached a plateau at a certain temperature, slightly varying from test to test, approximately at *T* = (350–465) °C. Initiation of TR took a duration of Δ*t*_TR_ = (51 : 57–73 : 50) min : s. With an increasing sample mass (Table 6) and only slightly increased heating power (Table 4), Δ*t*_TR_ increased significantly. There are only a few comparable experimental data for TR experiments on modules available in the literature (Section 1). Cheng *et al.* used an NMC module with *E* = 1.65 kW h for a TR test.^118^ The time to TR reported is Δ*t*_TR_ = (12 : 23–12 : 28) min : s. These lower Δ*t*_TR_ values were obtained with an entirely different initiation method, *i.e.*, two heating plates, each *P* = 400 W, were used to heat one of the twelve cells (*E* = 0.137 kW h per cell) placed in the middle of the module, with the aim to observe the propagation within the module. Thus, this type of initiation is better to be compared with tests #05 and #06 (Table 4: Heating power and Table 5: Δ*t*_TR_). In the test reported by Cheng *et al.*, the propagation was complete with the TR initiation of the last cell after Δ*t*_TR_ = 39 : 29 min : s,^118^ which is more in the range of test #07–#09 (Table 5). Comparable tests were carried out by Gao *et al.*,^30^ with NMC-modules (*E* = 1.625 kW h) consisting of twelve cells. TR was initiated at one side and the propagation was complete within Δ*t*_TR_ = 66 : 10 min : s.^30^ This value is similar to the Δ*t*_TR_ of tests #07–#09 (Table 5), where it should be noted that in both cases, initiation occurred from one external side of the module.

**Table 6:** Initial mass, sample mass after test and relative mass loss

Test no.	Initial mass [kg]	Mass after test [kg]	Relative mass loss [%]
#01–#04	0.26	Not evaluated	—
#05	0.78	0.37	53
#06	0.78	0.57	27
#07	13.0	7.49	42
#08	13.2	6.36	52
#09	12.7	2.30	82
#10	30.8	17.40	44
#11	30.9	7.44	76
#12	31.1	19.69	37

**Fig. 7:**
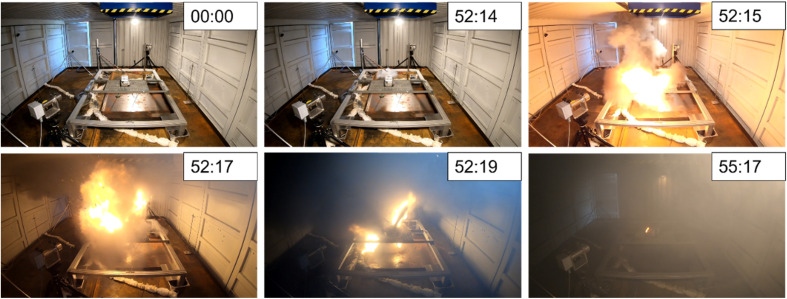
Time sequence of 2.65 kW h module in test #07 during TR with released gas and igniting jet flame.

The onset temperature was in the range of *T*_onset_ = (109–135) °C (*cf.*Table 5). The behaviour during TR of the modules differed from the cell tests, where before TR started, no strong pre-gassing or other signs of the upcoming event were observed. In test #07 (Fig. 7), no anomalies became visible until Δ*t* = 52 : 12 min : s. Only one second later, a gas jet was ejected, which immediately ignited and formed a jet flame. The strong exothermic reaction accompanying the jet flame lasted for Δ*t* ≈ 60 s, and then the reaction slowed down to an eased off burnup. During the reaction, the gaseous combustion products significantly decreased the visibility inside the experimental room.

**Fig. 8:**
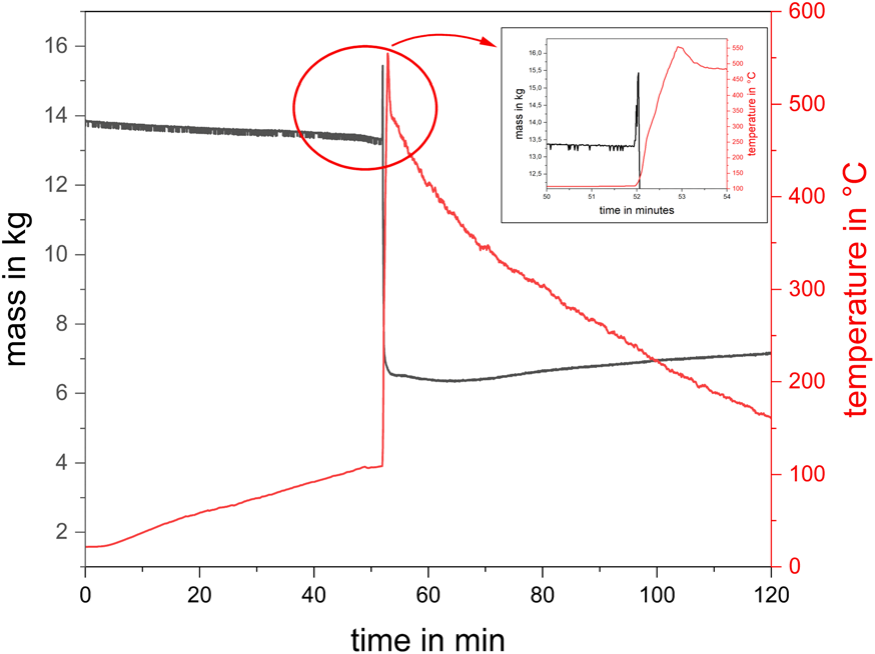
Temperature at the module top and mass over time for a 2.65 kW h module in test #07 and inset: load cell signal shows influence of released gas pulse.

Furthermore proof of the pronounced TR events is the high values for the mass burnup (Table 6). For instance, a mass burnup of Δ*m*/*m*_0_ ≈ 82% in test #09 was measured, *i.e.*, 10 kg. It must be considered that the mass difference did not solely result from the chemical reaction of the module components by TR. Part of the mass difference also occurred because of the opening of the aluminium case of the module and loss of some cell components, which were thrown outside. In test #09, the module itself was almost completely emptied. The experimental setup did not allow to distinguish between the fraction of mass lost due to the chemical reaction *versus* the fraction of mass lost due to parts thrown outside. A closer look to the load cell signal revealed that the momentum caused by the released jet flame could be identified. Fig. 8 shows the temperature on the top of the module and the load cell signal of test #07. At the beginning of TR, a high peak temperature was recorded. This is when the gas release started, and the weight signal of the module showed a sharp increase, which was caused by the impulse of the gas flow. Subsequently, the mass of the module decreased rapidly due to the escaping mass. Initially, the load cell signal decreased, and sometime later increased again. This is a temperature-compensation effect (first Δ*t* ≈ 50 min: heating process; after TR: cool down).

**Fig. 9:**
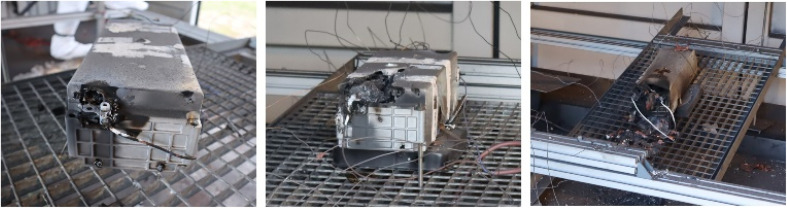
Damaged tested modules from: test #07 (left), test #08 (middle), and test #09 (right).

The maximum temperatures measured at the top of the modules varied from test to test. In test #07, *T*_max_ = 551 °C was reached, whereas in test #08, *T*_max_ = 947 °C, and in test #09, *T* = 332 °C were achieved. The maximum measured surface temperature in test #09 was *T*_max_ = 964 °C, which was measured at the bottom (Fig. 6). It should be noted that the modules were damaged differently, and sometimes even the thermocouples were torn off. As marked in Fig. 6, the thermocouples at the module top were at the initial measuring points in test #07 and #08 but not in test #09. The reason for this is depicted in Fig. 9, where the module of test #07 and #08 shows only minor to medium damage to the case and the thermocouples are at their initial measuring points. In test #09, the module case showed significant damage, it was wide open, and the top thermocouple was torn off. This was also the test associated with a high mass loss (Table 6). One reason for the significantly heavier TR in test #09 could be the higher initial voltage of the module (Table 3: *U*_#09_ = 25.4 V *vs. U*_#07/#08_ = 12.2 V) caused by another internal wiring.

#### 3.1.4 Large module tests

**Fig. 10:**
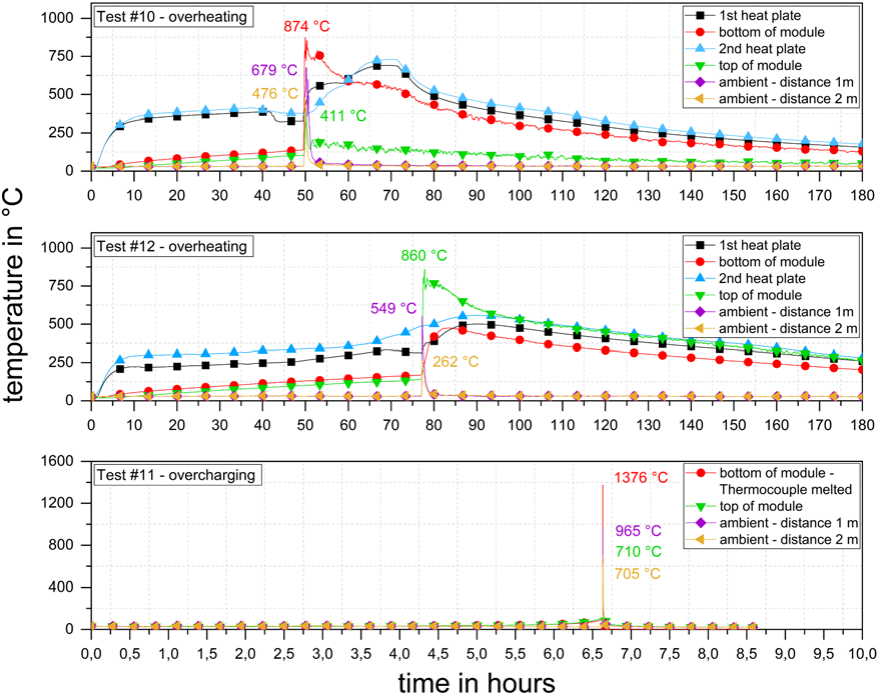
Temperature–time–histories of TR experiments with 6.85 kW h modules (test #10–#12) and temperatures of the heating plates (for #10 and #12), at the top and the bottom of the modules and at distances of *l* = (1 and 2) m.

Fig. 10 depicts the temperature–time–histories of the 6.85 kW h modules. In the overheating tests, #10 and #12, similar temperature curves were obtained. The time until TR occurred was Δ*t*_TR_ = 49 : 36 min : s in test #10 and Δ*t*_TR_ = 77 : 04 min : s in test #12. In test #12, a different internal wiring and the associated different voltages (Table 3; #10: 31.0 V and #12: 47.6 V) were set up, and thus a short Δ*t*_TR_ occurred. Slow, weak pre-gassing was observed, which lasted for Δ*t* ≈ 30 min. The other module overheating tests (#07–#10) did not show this type of behaviour (Section 3.3).

The onset temperatures *T*_onset_ = (123; 138) °C were similar to the onset temperatures of the smaller modules (Table 5). *T*_max_ primarily depends on the duration, location and intensity of the released jet flame. In the 6.85 kW h-tests, at least one temperature signal in each test remained above the threshold of *T* = 675 °C for longer than Δ*t* = 10 min. At about Δ*t* = 100 min after the start of TR, at least *T* = 250 °C prevailed on the surface of the module. In comparison, the 2.65 kW h module already cooled down to below *T* = 250 °C after 10 min. It can be stated that with a higher energy content of the modules, the duration of TR at a high temperature level increased. Initially, just a part of the larger modules reacted during the peak of TR and the reactions progressed more slowly. Further, the higher temperature maxima suggest higher heat release rates. In conclusion, for two of the smaller modules, stronger TR was detected. Moreover, the heat transfer was influenced by the dimensions of the modules.

**Fig. 11:**
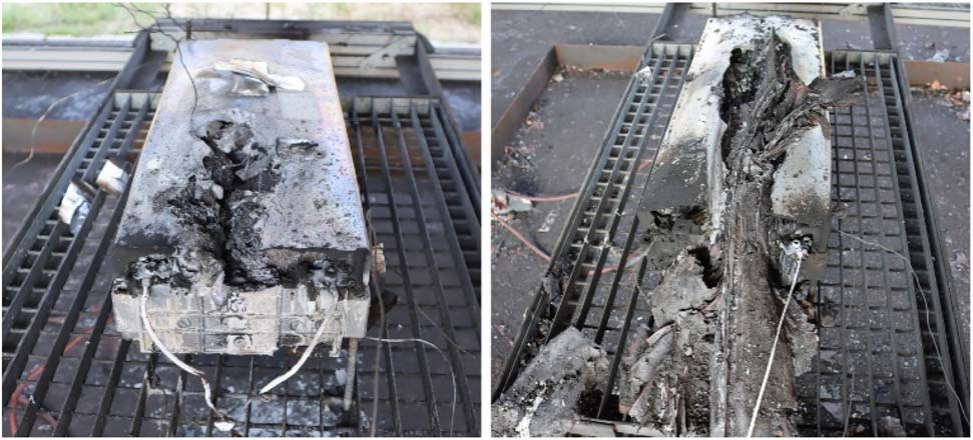
Damaged test modules from overheating test #10 (left) and overcharging test #11 (right).

The deviation in the maximum temperature at the surface of the modules during TR arose because the module was torn differently. However, in most cases, the initial formation of cracks occurred at the battery poles, which must be considered the mechanically weakest point under thermal stress. In test #10, the maximum temperature was *T*_max_ = 874 °C, which was measured at the bottom of the module. The maximum surface temperature at the top was *T*_max_ = 411 °C. In this test, the module was torn open, and there was a jet flame at the bottom. In contrast, in test #12, the maximum temperature of *T*_max_ = 860 °C was reached at the module top. Accordingly, this module was torn open on top side (Fig. 11, left).

Comparing the overheating tests of the large modules (#10 and #12) with the small module tests (#07–#09), it can be stated that a higher energy content did not necessarily lead to higher temperature maxima, while the 6.85 kW h modules led to *T*_max_ = (874; 860) °C, and the maximum measured temperature in the 2.65 kW h module-tests was about *T*_max_ = (551; 947; and 964) °C. Comparing these maximum temperatures with the results in the literature, values of *T*_max_ = 850 °C were reported for NMC modules (*E* = 4.15 kW h) in the experiments by Held *et al.*^113^

Generally, there was some general difference between the temperature–time–histories of overheating tests #10 and #12 in comparison to that of overcharging test #11. In the overcharging test, the module was stressed much less per unit of time than the modules stressed in the overheating tests. The overcharge rate in test #11 was very low at *Q* = 0.05C, which resulted from the module capacity of *C* = 207.5 A h and charge current of *I* = 10 A. Consequently, it took Δ*t*_TR_ = 398 min for TR to start and the reaction was more intense and violent than in overheating tests #10 and #12. The maximum temperature of *T*_max_ = 1376 °C was clearly higher than that in the comparable overheating experiments (Fig. 10). Due to these high temperatures, 3 out of the 10 thermocouples melted.

In tests #10 and #12, the mass burnup was Δ*m*/*m*_0_ = (36–44)%. Alternatively, in overcharge test #11, it was Δ*m*/*m*_0_ = 76%. This higher mass burnup was clearly visible in the damage of the module after the test (Fig. 11). Overall, the highest value for the mass burnup was found to be Δ*m*/*m*_0_ = 82% in overheating test #09.

#### 3.1.5 Heat sink before TR

**Fig. 12:**
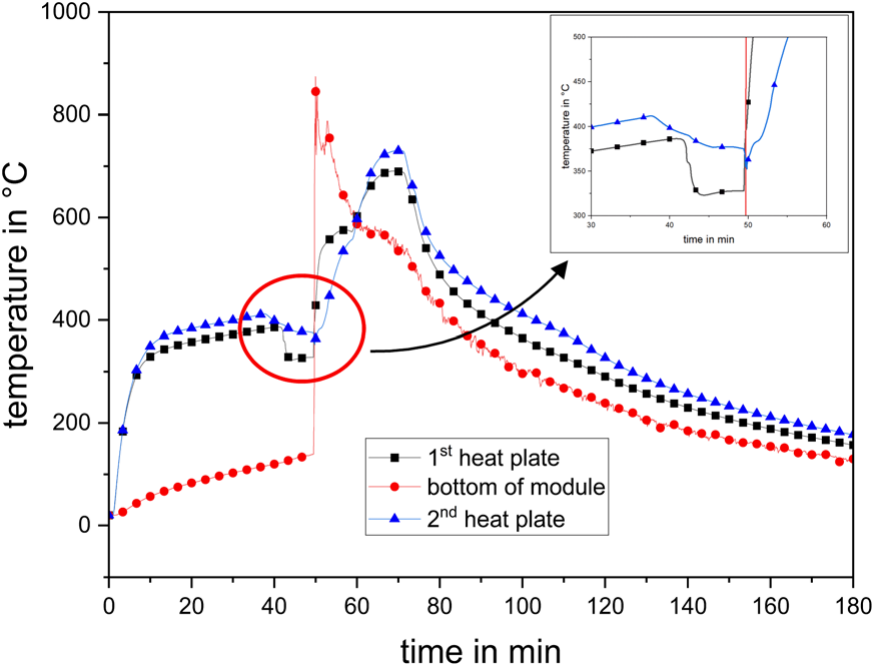
Temperature signal test #10, both heating plates and bottom of the module, and inset: temperature decrease before TR.

Although a constant heating power was applied, shortly before TR, a sudden drop in temperature was measured. Fig. 12 depicts the temperature–time–histories of test #10 as an example. In the final stage of the heating phase, *i.e.*, approximately Δ*t* = (5–10) min before Δ*t*_TR_, the temperature suddenly dropped. The temperature of the hot heating plate cooled down by Δ*T* = 80 K (#10 at *P* = 1300 W). At this time, no prior outgassing was visible for the modules except for test #12, where an internal process inside the modules required some thermal energy, which could be physical processes (*e.g.*, onset of evaporation of the electrolyte) or chemical reactions such as decomposition processes.

This heat consumption phenomenon has already been reported in the literature.^117,123,124^ Mao *et al.* observed a maximum temperature drop of Δ*T* = (29–35) K for a *C* = 300 A h prismatic LFP battery (*E* = 1100 W h, SOC = 100%) at a heating power of *P* = 500 W. On the one hand, the reason given for this phenomenon was that the previous outgassing of the cells/modules transported some of the vaporized hot electrolyte to the outside. On the other hand, the cell/module was under pressure, and thus depressurization caused cooling due to the Joule–Thompson effect.

### 3.2 Near field consequences

The release of chemical compounds, *i.e.*, heavy metals and poisonous gases, and the release of energy, *i.e.*, heat and flames, are the main effects of TR reactions (Section 1). Although high temperatures can be reached on the surface of the cells/modules, the effect on the near environment is determined by the total amount of energy and chemicals released. In the case of a single cell, this energy and chemical release were limited (Table 1: #01–#04), and thus a maximum temperature increase of Δ*T* = 4 K was detected in the nearfield (*d* = (1 and 2) m). However, this was not a sudden increase at the time of TR but a general increase over the entire test period. The distinction between the effect of the reaction energy release and the changing weather conditions is open. The impact of temperature on the near environment in the case of TR in a single cell is negligible, which is probably slightly higher than lighting a match. However, this situation changes significantly in the case of cell stacks and modules, respectively.

Table 7 presents the temperature increase for the tests with cell stacks and modules. In the case of the cell stacks, the measured maximum short-term temperature increase was in the range of Δ*T* ≈ 10 K. It should be noted that this temperature peak at the TR was measured by thermocouples convoluting the true temperature with a certain signal lag time. The heat impact on the environment in the cell stack experiments was small but significant, and thus not negligible.

**Table 7:** Maximum ambient temperature increase at *l* = (1 and 2) m, with the maximum value underlined for each test in both distances

Test no.	Δ*T* [K], north, 1 m	Δ*T* [K], west, 1 m	Δ*T* [K], south, 1 m	Δ*T* [K], east, 1 m	Δ*T* [K], north, 2 m	Δ*T* [K], west, 2 m	Δ*T* [K], south, 2 m	Δ*T* [K], east, 2 m
#05	7.0	6.6	**8.7**	6.1	7.1	9.3	8.9	**9.8**
#06	5.6	5.2	**7.7**	4.8	4.9	8.2	6.7	**8.5**
#07	178.4	**271.5**	241.4	147.4	253.5	**360.1**	228.0	188.8
#08	123.7	122.9	**155.9**	117.0	191.1	186.6	**211.6**	189.8
#09	193.3	196.2	**657.6**	322.1	317.4	265.6	**832.4**	437.2
#10	215.0	149.3	**650.1**	183.2	248.7	317.5	**447.9**	289.0
#11	217.0	280.1	**934.2**	192.1	**674.7**	395.9	450.8	393.5
#12	63.4	47.5	**521.3**	53.2	88.0	114.4	**235.0**	129.3

In the case of the 2.65 kW h module tests, a maximum temperature increase of Δ*T* = 832 K was measured. The temperature was higher at a distance of *l* = 2 m, and then *l* = 1 m (Table 7: test #09). The temperature sensors were placed at different heights (*l* = 1 m: *h* = 0.5; *l* = 2 m: *h* = 1 m). The higher measuring points were more affected by the convective flow of heat. In the case of the overheating tests of larger modules (Table 1: test #10 and #12), the maximum temperature increase was Δ*T* = 650 K. The highest Δ*T* value was observed in the overcharging test (Table 1: test #11). At a distance of *l* = 1 m, a temperature increase of Δ*T* = 934 K was detected.

Comparing the different initiation methods, overheating (test #10 and #12) and overcharging (test #11), it can be concluded that overcharging can produce a higher thermal effect than overheating. The significantly stronger impact on the environment during TR of lithium-ion batteries triggered by overcharging instead of overheating is in good agreement with the experiments by Huang *et al.*^98^ In these experiments, NMC cells each with *E* = 160 W h were investigated. TRs were initiated in the overheating tests at an SOC of 100% with a heating power of *P* = (300 and 400) W and in the overcharging tests with charging rates of *Q* = (1 and 0.5)C. The results show that a stronger temperature effect on the environment is found in the HRR. The overcharging experiments resulted in an HRR of *Q̇* = 70.4 kW at *Q* = 1C, whereas the overheating experiments at *P* = 400 W only led to a heat release of *Q̇* = 11.9 kW.

According to Table 5, the highest temperature increase was often in the south orientation. This is because a north-south longitudinal orientation was specified by the attachment and mounting of the modules on the sample table. Given that most of the modules were opened during the TR on the pole side (south direction) with a jet flame, the highest values were also found there. However, an exception in this context is test #07. This module was not mechanically fixed, and thus rotated by an angle of 135° (Fig. 7). This movement and the following different thermal effect on the surrounding deviated from the other tests, and thus the temperature data in Table 7 shows the highest temperatures in the west direction.

**Fig. 13:**
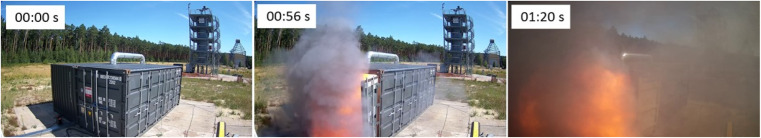
Test #09, subsequent gas explosion due TR and pressure release of the experimental room.

The TR in all the tests was accompanied by a jet flame. In the case of the cell tests, both single and stacks, the length of this jet flame was usually *l* = 50 cm in a nearly horizontal direction (Fig. 5). In the case of the modules, overheating jet flames of *l* = (2–3) m were observed. In most cases, the jet flame was 45°–60° in the upper direction (Fig. 7). As already mentioned, in addition to overcharge test #11, the TR effects were also particularly severe in test #09. In addition to overloading in test #11, the consequences of TR were also severe in test #09, as already noted above (*cf.* mass burnup Δ*m*/*m*_0_ = 82% and temperature increase in surrounding Δ*T* = 832 K). The reason for this violent reaction behaviour in test #09 is the fast, explosive TR, followed by a gas explosion, as reported in the literature.^26^ The first sign of TR was the jet flames in the pole section, directly followed by massive gas release. Within Δ*t* = 2 s, the whole experimental room was filled with smoke gas, followed by a gas explosion. The violent reaction of the module in test #09, from the first sign of TR to the end of the subsequent gas explosion, was finished within Δ*t* = 5 s. During this reaction time, most of the “inside” of the module was ejected to the outside (Fig. 9, right), followed by a slowly flattening burnout of the residual parts of the module. The explosion pressure abruptly opened the side door of the test room, which was left ajar, and a few tenths of a second later, the pressure release was complete. This sequence of events is depicted in Fig. 13. Tests #07–#09 took place under almost identical initial conditions, but the voltage of the module in test #09 was twice that of the other tests (Table 3: test #09: *U* = 25 V; test #07 & #08: *U* = 12 V). This could be one explanation for the different behaviours.

The TR in test #09 resulted in a violent explosion, and thus in the subsequent tests #10–#12, the FTIR sampling equipment and the DLS were removed from the experimental room (exception: DLS in test #12). The little extra possible knowledge potentially gained by this parameter was believed to be insignificant in comparison to the costly equipment.

In these tests, which were accompanied by high temperatures and jet flames, hot metal dust was emitted, and fragments were ejected, flying through the experimental room. Thus, a high amount of mass was lost during the tests, *i.e.*, due to being ejected (metal parts), being evaporated (electrolyte) or oxidized (chemical reaction). The surfaces inside the experimental room were completely covered with dust after the tests. Partly, several millimetres of dust covered the floor of the experimental room. The fire residues in conjunction with water had a pH value of approximately 11. The destroyed modules are shown in Fig. 9 and 11. Heavy metals (*e.g.*, nickel, manganese, and cobalt), lithium and graphite were emitted as dust from the jet flame, which led to the contamination of the experimental room. The toxic effect and the load on clothing and surfaces was investigated by Held *et al.*,^113^ among others. They also used NMC cells and obtained similar results, with pH = 12.3.

**Fig. 14:**
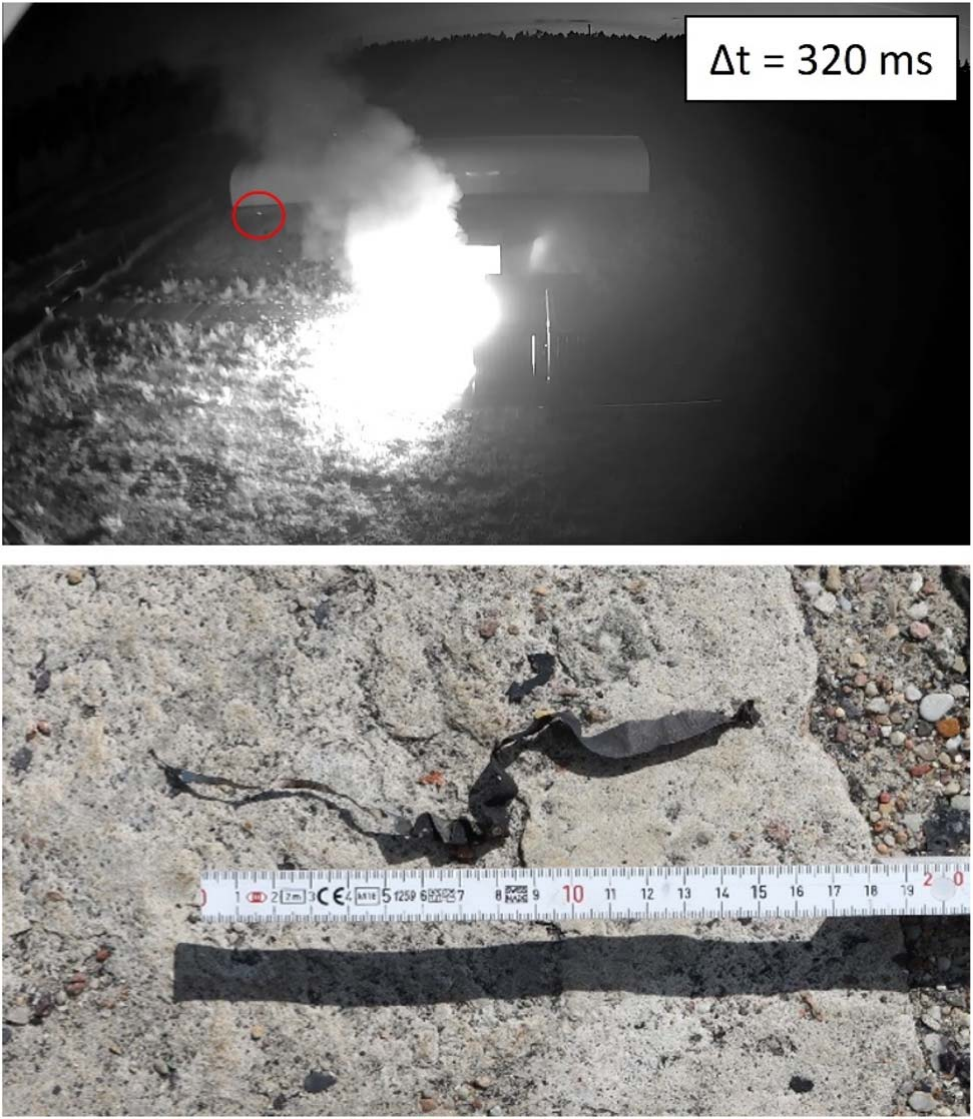
Test #11, top: picture taken from a video recorded in night vision mode, Δ*t* = 320 ms after TR, flyaway fragment red circled, bottom: fragment, length *l* = 17 cm found at a distance of *l* ≈ 33.2 m.

In certain tests, some fragments flew though the open doors. This indicates that initially the doors opened due to the overpressure generated, and secondly the fragments were ejected. Module test #11 is depicted in Fig. 14. The burning/glowing metal fragments are visible in the video recorded in night mode (Fig. 14, top). Smaller metal parts were found in close proximity, about *l* = (5–10) m in front of the experimental room. Parts with larger mass and momentum were found at the end of the access road (Fig. 14, top: circled in red). One of these fragments, length *l* = 17 cm, was found at a distance of *l* ≈ 33.2 m (Fig. 14, bottom). This demonstrates that hot metal pieces were ejected, with a certain probability of initiating subsequent fires. This is the most relevant in environments with a highly flammable fire load (dry grass or dry wood may be sufficient).

### 3.3 Gas release

#### 3.3.1 Visibility

Each cell/module TR was usually accompanied by gas release, followed by a flame of various intensities (Section 3.1). An exception is cell test #04, where the TR was only characterized by gas release. The time span between the point where the gas release was first visually perceptible up to the appearance of a flame was analyzed (test #04: due no flame occurs, time span from slightly beginning to strong gushing gas release). For the single cell tests (*cf.*Table 1: #01–#04), this time span is in the range of Δ*t* = (77–204) s. For the tested cell stacks (*cf.*Table 1: #05 and #06), the time span for the TR of the first, lowest cell is similar, Δ*t* = (55 and 139) s. In the cell tests, the preliminary lead time from the first visual sign of the beginning TR to the main exothermic reaction was sometimes less than one minute.

Completely contrary and inhomogeneous observations were made in the six module tests. In test #12, a 6.85 kW h module, a prolonged outgassing phase started at Δ*t* = 1856 s, lasting for more than half an hour before TR began and a flame was visible. Three other tests were characterized by a short phase of slightly visible gas release before TR. This was comprised of small module tests #08 (Δ*t* = 161 s) and #09 (Δ*t* = 49 s) as well as the large module overcharging test #11 (Δ*t* = 135 s). The two remaining tests showed extremely short outgassing phases. This applies to both test #07 with Δ*t* = 3 s and test #10 with Δ*t* = 5 s. For test #07, this is depicted in Fig. 7. In both tests, the exothermic reaction started with nearly no warning. During TR, the room filled with smoke rapidly (Fig. 5 and 7). The colour of the released gas varied from white at the beginning of the reaction (Fig. 5) to grey/darker when the reaction became more violent (Fig. 7 and 13). In this case, the darker colour is probably due to the limited oxygen content in the closed/half-closed experimental room and suggests that the oxidation reaction was incomplete. The darker colour of the smoke may also be due to the increasing release of dusty graphite. Other changes in the colour of the released gas, such as that reported in the literature,^118^ were not detected.

#### 3.3.2 Hydrogen fluoride (HF)

**Fig. 15:**
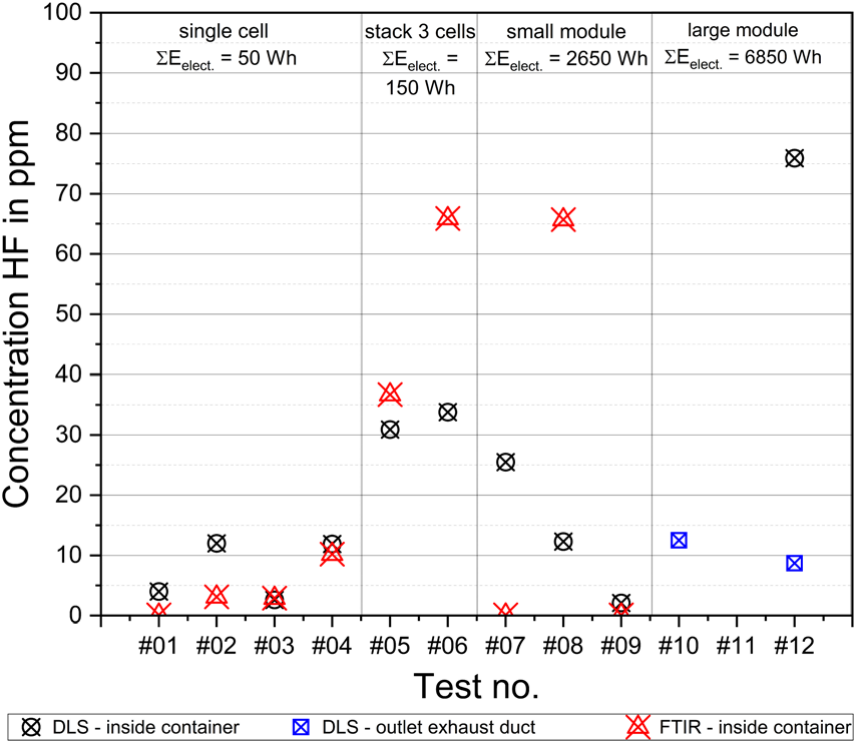
Results for the HF measurement with two different systems: FTIR and DLS.

Two different measurement systems (FTIR and DLS, Section 2.2) were used for HF measurement. Both systems were used from test #01–#09. Additionally, the DLS for HF was used in the experimental room in test #12 and a second system at the end of the exhaust line in tests #10 and #12. The maximum HF concentration measured in each test is summarized in Fig. 15 for both systems.

The HF values in the single cell tests (#01–#04, Table 1) are in the range of *c*_HF_ = (2.6–12.0) ppm, which were measured with the DLS. The equivalent values from the FTIR measurement are in the range of *c*_HF_ = (2.9–10.2) ppm, excluding test #01, in which no HF was detected. When comparing the measured values of the two systems, they show good agreement, especially in tests #03 and #04. In test #02, the DLS-value is four-times higher than the measured FTIR-value.

The HF concentration generated during the TR was significantly higher in case of the cell stack tests. In test #05, the concentration measured with FITR was *c*_HF FTIR_ = 36.7 ppm, while that for DLS was *c*_HF DLS_ = 30.9 ppm. Second stack test #06 resulted in *c*_HF FTIR_ = 65.9 ppm and *c*_HF DLS_ = 33.8 ppm, respectively. An increase in HF emission with an increasing cell number was reported by Sturk *et al.*^61^ for LFP cells, which was also confirmed to be valid for NMC cells. However, only a few data is available on TR in other batteries in the literature concerning measured HF concentration. Instead, the total amount of emitted HF is often stated (*e.g.*, in g) or the amount is related to the energy content of the test samples (*e.g.*, in g W^−1^ h^−1^).^20,85,86^ Indeed, the indication of the values in this manner is the only way to compare tests from different literature reference.^125^ The experimental data presented in Fig. 15 are given in concentration, not in total amount released. The determination of the total amount released HF was not possible with the experimental setup used. Another obstacle in realizing a comparison is the large variety of cells tested in the literature (Section 1). The various parameters are the energy content, cell geometry, cathode material, and SOC. For example, Larsson *et al.*^60^ indicated values for HF concentrations from TR-tests with LFP pouch cells. Due to the different cathode materials, a comparison of the results has limited significance. It can only be stated that the magnitudes of the measured concentrations agree, with *c*_HF_ = (140–150) ppm,^60^ (2 LFP pouch cells, *C* = 40 A h, SOC = 100%) *versus c*_HF_ = (30.9–65.9) ppm (Fig. 15, 3 NMC pouch cells, *C* = 37.5 A h, SOC = 100%). Huang *et al.* measured a concentration of *c*_HF_ ≈ 80 ppm in a test with NMC cells. However, it must be considered that the cells used had quite a higher energy content (4 cells, each *C* = 100 A h) and different cell geometry (prismatic) compared to the test results in Fig. 15.^102^ Consequently, it can also only be stated here that the decimal magnitude fits.

The module tests (Fig. 15: #07–#10 and #12) show no further increase in HF emission. Lecocq *et al.* showed that for LFP cells with increasing SOC, the emitted HF decreased. This is attributed to the decomposition of the salts in the electrolyte, which is promoted by the prolonged fire duration of the lower SOC cells.^79^ Subsequently, an extensive exothermic and fast reaction, such as in the small module tests (Fig. 7: main reaction of a module with *m* ≈ 13 kg ends within Δ*t* ≈ 3 min), will lower the HF emission. This can be confirmed by the results shown in Fig. 15. This is also well matched by the fact that the TR reaction in test #09 was particularly severe (including the subsequent explosion, Section 3.2 and Fig. 13) and nearly no HF was measured with both systems (*c*_HF DLS_ = 2.1 ppm and *c*_HF FTIR_ = 0.1 ppm).

The values from DLS and FTIR in test #07 and #08 differ more than in the cell level tests. In test #07, the concentration measured with DLS was *c*_HF DLS_ = 25.5 ppm, whereas with the FTIR, only an extremely low HF concentration was detected (*c*_HF FTIR_ = 0.1 ppm). In test #08, the measured values show the opposite behavior with a five-fold concentration detected with FTIR *versus* DLS (*c*_HF DLS_ = 12.3 ppm *versus c*_HF FTIR_ = 65.7 ppm). One reason for this is the different measuring principles. DLS is an optical method, which is measured on a line. In contrast, the FTIR measurement is only at one point, *i.e.*, the suction point of the sampling lance (Fig. 2). In the cell tests (#01–#06), this different measurement principle does not have much importance because the measurement is performed in the same region with simultaneous minor convection due to the manageable reaction in the relatively large experimental room. In contrast, the reaction in the module test was much more severe, which is associated with a clear higher movement of the releasing gas (Fig. 7 and 13). This leads to the fact that the different measurement principles and small deviations in the measurement location become much more important. The measurement is also influenced by the required transmission of the DLS (Section 2.2: *τ*_min_ ≥ 1%). Due to the large amount of released gases in the module tests, the transmission was influenced significantly more than in the smaller cell tests. This caused the transmittance to drop to *τ* < 1%, and thus the measurement was no longer possible. Due to the test conditions outside the usage limits of the DLS, a potential higher HF value was not detected using this measuring device.

According to the tests performed thus far, knowledge about the vent gas composition was available, and thus in big module tests #10–#12, FTIR was not applied as well as DLS inside the experimental room in tests #10 and #11. Obviously, high concentrations of highly poisonous compounds could have been measured; however, considering chemical safety engineering, this would lead to no further gain in knowledge with complete destruction of the analytical instrument.

The measured concentration in test #12 showed the highest amount among the tests, with *c*_HF DLS_ = 75.9 ppm. This means a slight increase compared to the highest measured concentrations in the small module and cell stack tests. The fact that a higher amount was measured and not a lower value as in most cases in the small module tests is probably due to the fact that test #12 was characterized by a significantly longer outgassing phase before TR started (Section 3.3, Visibility: outgassing before TR of Δ*T* > 30 min). At the end of the unheated exhaust line, a concentration of *c*_HF_ = 8.7 ppm was measured in this test and *c*_HF_ = 12.5 ppm in test #10.

Another mentionable aspect is the detected HF concentrations before TR occurred. In this case, all concentration–time-sequences of the DLS were categorized with respect to two different threshold values, *i.e.*, *c*_HF_ = (1 and 10) ppm, as well as their temporal occurrence before TR. In most of the tests, the 1 ppm-threshold was reached minutes before the TR criteria were fulfilled (Section 3.1), *e.g.*, two of four single cell tests with Δ*t* ≈ 120 s or the cell stack tests within Δ*t* = (107 and 111) s before TR. Similarly, in two of the three small module tests, this threshold was reached about Δ*t* = 133 s (test #08) and Δ*t* = 370 s (test #09) before the TR.

A particularly early HF vent gas concentration was measured in test #12, which is the only large module test with the use of DLS. Here, the 1 ppm-threshold was reached Δ*t* = 1118 s before TR and the 10 ppm-threshold was still about Δ*t* = 158 s. The results of test #12 fit very well with the prolonged outgassing phase described above (Section 3.3, Visibility). It can be stated that in several tests, a low HF concentration was already measured before the TR and before any visible outgassing.

For a better understanding of the measured HF concentration and its toxic effects on humans, a comparison with established risk level classifications is appropriate, *i.e.*, AEGL (acute exposure guideline levels) and IDLH (immediately dangerous to life and health). The HF AEGL-2 for disabling is stated at *c*_HF_ = 34 ppm within an exposure time of Δ*t* = 30 min and *c*_HF_ = 95 ppm within Δ*t* = 10 min.^126^ The 30 minute-concentration was fulfilled in both the module tests and cell stack tests. The 10 minute-concentration was not reached. The same applies to the AEGL-3 for lethal impact. The level was reached for the 30 min-threshold (*c*_HF_ = 62 ppm) but not for 10 min-level (*c*_HF_ = 170 ppm).^126^ The IDLH level is defined as the threshold for immediately or delayed permanent health effects or causes death. The threshold for HF is stated to be *c*_HF_ = 30 ppm.^127^ This value was reached also in both the module and cell stack tests.

#### 3.3.3 Multi gas analysis

**Fig. 16:**
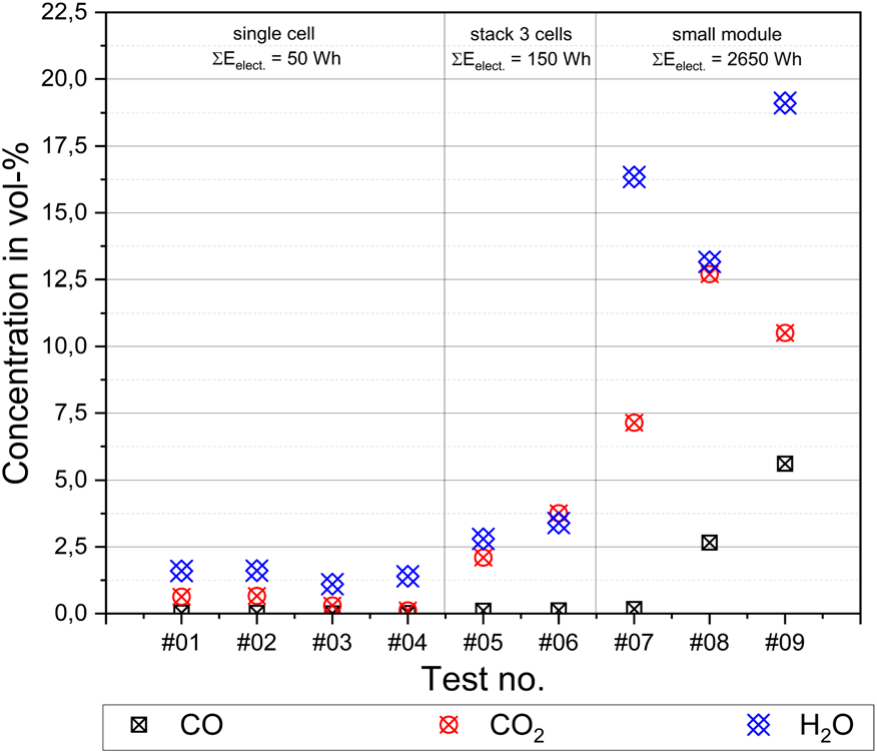
FTIR measurement results of CO_2_, H_2_O and CO for tests #01–#09.

The data presented are the results from the FTIR measurement in tests #01–#09. The main reaction products of complete combustion are CO_2_ and H_2_O, as well as CO for incomplete combustion. The data measured for these nine tests are depicted in Fig. 16. With an increasing energy content of the test sample, the amount of combustion products increased. The highest concentrations were detected in test #09, *i.e.*, for H_2_O (*c*_H_2_O_ = 19.1 vol%) and CO (*c*_CO_ = 5.6 vol%), and for CO_2_ in test #08 (*c*_CO2_ = 12.7 vol%). The higher the amount of combustion products, the larger the energy content released and the size of the test samples. Alkanes (CH_4_, C_2_H_6_, C_3_H_8_, and cyclohexane) and alkenes (C_2_H_4_ and C_3_H_6_) were detected in a range of some hundreds of ppm at maximum in almost all the different tests presently reported. The only exception in this context was test #08, with *c*_alkanes_ = 4169 ppm and *c*_alkenes_ = 6460 ppm.

Test #09 was characterized by a subsequent explosion of the released gas (Fig. 13). The measured data (Fig. 16) indicates there was a mixture of some combustible gases present prior to explosion. CO was one of these gas compounds. The lower flammability limit (LFL) for CO is stated to be *c*_LFL CO_ = 10.9 vol% under atmospheric conditions.^128^ Because the gas sample for FTIR analysis was taken at a certain point, even some higher concentrations than measured could have been present. It should be noted that a homogeneous mixture was absent inside the experimental room. The flammable gases released were alkanes, alkenes and CO, as well as hydrogen, as reported in the literature.^71^ However, the hydrogen molecule is not visible in IR. Due to the small concentrations of alkanes and alkenes, as stated above, these components only have a small influence on the LEL of a gas mixture with *c*_CO_ = 5.6 vol%. The LEL for methane is stated to be *c*_LEL CH_4__ = 4.4 vol% and for ethane c_LEL C_2_H_6__ = 2.4 vol%.^128^ However, alkanes, alkenes and hydrogen have one thing in common, which is that they have an LEL much lower that of CO. Subsequently, the calculated LEL from a gas mixture of CO, H_2_, alkanes and alkenes according to the mixing rule of Le Chatelier will be in all cases lower than of CO alone. Maloney calculated the LEL for a vent gas of LCO-cells in a range of *c*_LEL_ ≈ (8–9) vol%.^108^ Consequently, the conclusion can also be drawn that in particular, in experiment #09 with the subsequent explosion, due to local inhomogeneities, higher proportions of alkanes and alkenes than that measured were present and/or a significant proportion of H_2_. Larger amounts of H_2_, alkanes and alkenes as vent gas proportions of NMC cells were recently reported in the literature.^63,70,71^ In this context, it can be stated that the experimental setup used is suitable for qualitative conclusions on the released gases. The quantitative measurements presently reported were limited to a single sample point inside the experimental room. Thus, the total mass release of a certain compound could not be deduced from this inhomogeneous sample probe. However, concentration peaks could be identified. Different experimental setups (*e.g.*, closed apparatus for TR experiments) and tests by other researchers aiming at the measurement of the total mass release of critical compounds already provide this type of data in the recent literature.^63,70,71,77^

The measured sum of carbonate ester (EC, EMC, DMC, and DC) is in the range of *c*_CE_ = (0.08–0.29) vol% in test #08 *c*_CE_ = 0.69 vol%. POF_3_ was also measured in the NMC cell tests reported by Wang *et al.*^92^ and LCO-cells by Larsson *et al.*^60^ POF_3_ was detected in all of the nine tests, with a range of *c*_POF_3__ = (8–62) ppm. A direct comparison of the concentrations is difficult because of the different experimental setups aimed at the measurement of released species as total mass^92^ and mass release rate,^60^ respectively.

The measured range of methanol and ethanol in total is about *c*_alcohols_ = (33–522) ppm in test #07, with *c*_alcohols_ = 1221 ppm. Small amounts of hydrogen chloride were also detected in all the tests. The concentration range was *c*_HCl_ = (0.5–19) ppm. The HCl AEGL-2 for disabled persons is *c*_HCl_ = 43 ppm at an exposure time of Δ*t* = 30 min and *c*_HCl_ = 100 ppm within Δ*t* = 10 min, respectively. According to the measured concentrations, these thresholds are clearly not reached, in particular the AEGL-3 concentration for lethal impact (30 min-threshold: *c*_HCl_ = 210 ppm).^126^ However, it must be considered that the atmosphere in the experimental room was inhomogeneous, and thus it cannot be ruled out that higher HCl-concentrations existed locally. In general, HF is extremely caustic and toxic, and thus the more critical gas released compared to HCl. Depending on the concentrations measured, the statement that it is HCl, and not HF, which governs the toxicity consideration,^129^ cannot be confirmed in this series of tests.

CO is also a critical compound when evaluating the toxicity of the released gases. Table 8 presents the corresponding AEGL-2 and AEGL-3 exposure times.^130^ Obviously, even in the cell tests, disabling effects are possible within Δ*t* = 30 min exposure time. The maximum measured concentrations in the cell stack tests can be affected lethal impact within Δ*t* = 30 min and in the module tests within Δ*t* = 10 min. Based on the AEGL, the measured maximum concentrations for CO are in most cases significantly more toxic than that for HCl and HF.

**Table 8:** Max. measured CO-concentration, corresponding exposure time for AEGL-2 for disabling impact and AEGL-3 for lethal danger^130^

Test no.	*c*_CO_ [ppm]	AEGL-2 exposure time [min]	AEGL-3 exposure time [min]
#01	303	30	240
#02	227	30	240
#03	57	240	—
#04	172	30	240
#05	1082	10	30
#06	1206	10	30
#07	1721	10	10
#08	26 627	10	10
#09	56 035	10	10

## 4. Conclusions

Overall, twelve TR experiments from single cell to 6.85 kW h module were performed under comparable initial conditions. All tested cells/modules underwent TR. The focus of these experiments was to evaluate the consequences on the surrounding. The key findings are as follows:

• The maximum measured thermal load of the module TR was Δ*T* = 934 °C at a distance of *d* = 1 m and Δ*T* = 832 °C at a distance of *d* = 2 m.

• A jet flame could occur in a distance range of *d* = (2–3) m due the heating-induced module TR and *d* = (4–5) m in the case of an overcharge-induced module TR. In most cases, the jet flames occurred in an upward angle of 45°–60°. The duration of the jet flames was about Δ*t* ≈ 60 s in the module tests.

• The highest probability of the direction of the jet flame and the highest thermal load on the surrounding were in the orientation of the module poles, *i.e.*, the weakest point of the case.

• A subsequent gas explosion of the vent gas could occur, considerably increasing the thermal stress and pressure stress on the surrounding.

• In most module tests, small metal parts were ejected. Their throwing distance was *d* = (5–10) m from the tested modules. In the overcharging experiment, the distance was *d* = 33.2 m for a metal part of *l* = 17 cm length.

• HF was detected in all the tests, whenever a measurement was performed. The maximum detected HF concentration was *c*_HF_ = 76 ppm. Both in the cell stack and module tests, the 30 min-AEGL-2 (disabling) and 30 min-AEGL-3 (lethal impact), *i.e.*, defined for 30 min of exposure, were reached.

• The HF concentration increases with increasing energy content of the test sample, especially comparing the single cell and the cell stack or module tests. In some cases, no difference in the amount could be detected between the module and the cell stack tests.

• When assessing the toxicity of vent gases, the focus must be on CO, in addition to HF. In the cell stack and module tests, concentrations of *c*_CO_ > 1000 ppm were measured, *i.e.*, up to *c*_CO_ = (2.6 and 5.6) vol%.

The findings presently reported should become a basis for the holistic risk assessment of stationary energy storage systems made from second-life modules. The information concerning the thermal load on the surrounding area and the jet flames (length and direction) are important aspects in the dimensioning of the fire-resistance duration of building components and building materials in constructional fire protection. The toxicity of the vent gas released is important for strategies of firefighting as well as for hazard concepts with respect to occupational health and safety. There is an unjustified focus on the HF concentration in the vent gas, but the CO concentrations are high, and thus must considered when assessing the toxicity of the released gas mixture for humans. The findings on fragments being ejected and the pressure effect of a subsequent gas explosion are particularly important for measures in the field of process and plant safety.

Concerning the initiation of the TR and the behavior of the samples during and after the TR, it should be stated that:

• The time to TR in the case of overheating as the initiation method was in the range Δ*t*_TR_ = (49 : 36–77 : 04) min : s for the tested modules.

• An extended time to TR in the case of module overload is possible. In one test, it was Δ*t*_TR_ = 398 min. This was due to the extremely low charging rate (*Q* = 0.05 C).

• Onset temperatures in the range of *T*_onset_= (109–138) °C were determined for the modules.

• The mass burnup of the modules was in the range of Δ*m*/*m*_0_ = (37–82)%. It should be that the overcharge test resulted in a high mass burn-up of Δ*m*/*m*_0_ = 76%. The most pronounced mass loss (Δ*m*/*m*_0_ = 82%) was achieved in a small module test with overheating initiation.

• For the tests with the 6.85 kW h modules, a remarkable difference was found in the peak temperature measured at the surface of the module between overcharging and overheating. Overheating led to Δ*T* = (860 and 874) K, while overcharging led to Δ*T* = 1376 K (exceeding the measurement range, melting the thermocouples). This is a difference of about 500 K.

When comparing the different initiation methods, it has been widely reported in the literature that the overcharging of cells leads to more severe effects than TR induced by heating. This was also partially confirmed herein for the tested modules, especially with regard to the maximum temperatures at the modules, the length of the jet flame or the fragment throw distances. However, under certain conditions (*e.g.*, in combination with a subsequent vent gas explosion), the overheating initiation method can also lead to comparable effects. Examples of this are the high mass burn-up and high temperatures affecting the surrounding area, even at a distance of *d* = 2 m.

The HF measurement with two systems (DLS and FTIR) revealed interesting information concerning the pre-gassing phase, as follows:

• The visually perceptible pre-gassing for the cells starts in the range of Δ*t* = (55–204) s before TR. In the module tests, this behavior varies widely, from more than half an hour in one test to two tests with Δ*t* < 5 s between the first visible outgassing and the start of the TR.

• Both HF measurement systems, FTIR and DLS, have advantages. With FTIR, it is possible to measure independently of the optical smoke density. Alternatively, with DLS, line measurement can be carried out, averaging over a certain distance, whereas with FTIR, only a point measurement is possible.

• An HF concentration of *c*_HF_ ≥ 1 ppm was reached in most tests at about Δ*t* = (100–140) s before TR. In two module tests, the 1 ppm-threshold-value even exceeded in the time of Δ*t* = (370 and 1118) s before TR.

It can be stated that the combination of the two HF measurement systems provided a comprehensive picture of the HF emission. Especially, in some module tests with a very short pre-gassing phase, the HF measurement *via* DLS can be used as early detection for outgassing and a precursor for the upcoming TR due to its sensitivity.

## Author contributions

Conceptualization, R. T., S.-K. H. and U. K.; methodology, C. B., C. L., R. T and S.-K. H.; software, C. B., C. L. and P. W.; validation, C. B., C. L., P. W., R. T. and T. R.; formal analysis, C. B., P. W. and R. T.; investigation, C. B., C. L., P. W. and R. T.; resources, C. B., P. W., R. T. and T. R.; data curation, C. B. and R. T.; writing–original draft preparation, C. B. and R. T.; writing–review and editing, C. L., P. W., S.-K. H., T. R. and U. K.; visualization, C. B. and R. T.; supervision, R. T., S.-K. H. and U. K.; project administration, R. T., S.-K. H. and U. K.; funding acquisition, R. T., S.-K. H. and U. K.; all authors have read and agreed to the published version of the manuscript.

## Conflicts of interest

The authors declare that no conflict of interest exists.

## Supplementary Material

## References

[cit1] IEA , Grid-Scale Storage, International Energy Agency, Paris, 2022, https://www.iea.org/reports/grid-scale-storage

[cit2] EIA, Battery Storage in the United States , An Update on Market Trends, U.S. Energy Information Administration, Washington, D.C., 2021, https://www.eia.gov/analysis/studies/electricity/batterystorage/pdf/battery_storage_2021.pdf

[cit3] ENER , Study on energy storage - Contribution to the security of the electricity supply in Europe, Directorate-General for Energy, Directorate B — Internal Energy Market, Brussels, 2020

[cit4] Wüllner J., Reiners N., Millet L., Salibi M., Stortz F., Vetter M. (2021). Curr. Sustain./Renew. Energy Rep..

[cit5] Zhao Y., Pohl O., Bhatt A. I., Collis G. E., Mahon P. J., Rüther T., Hollenkamp A. F. (2021). Sustainable Chem..

[cit6] Jindal P., Bhattacharya J. (2019). J. Electrochem. Soc..

[cit7] Hildebrand S., Friesen A., Haetge J., Meier V., Schappacher F., Winter M. (2016). ECS Trans..

[cit8] Kriston A., Kersys A., Antonelli A., Ripplinger S., Holmstrom S., Trischler S., Döring H., Pfrang A. (2020). J. Power Sources.

[cit9] Ouyang D., He Y., Chen M., Liu J., Wang J. (2018). J. Therm. Anal. Calorim..

[cit10] Morones A. (2022). Process Saf. Prog..

[cit11] Marlair G., Lecocq A., Bordes A., Christensen P., Truchot B. (2022). Chem. Eng. Trans..

[cit12] Blum A. F. and Long Jr R. T., Fire Hazard Assessment of Lithium Ion Battery Energy Storage Systems, Springer Nature, New York, 2016

[cit13] Abd-El-Latif A. A., Sichler P., Kasper M., Waldmann T., Wohlfahrt-Mehrens M. (2021). Batteries Supercaps.

[cit14] Börger A., Mertens J., Wenzl H. (2019). J. Energy Storage.

[cit15] Feng X., Zheng S., He X., Wang L., Wang Y., Ren D., Ouyang M. (2018). Front. Energy Res..

[cit16] Liu J., Wang Z., Bai J., Gao T., Mao N. (2022). Appl. Therm. Eng..

[cit17] Huang P., Yao C., Mao B., Wang Q., Sun J., Bai Z. (2020). Energy.

[cit18] Liu J., Wang Z., Gong J., Liu K., Wang H., Guo L. (2017). Materials.

[cit19] Ping P., Wang Q., Huang P., Li K., Sun J., Kong D., Chen C. (2015). J. Power Sources.

[cit20] Larsson F., Andersson P., Blomqvist P., Lorén A., Mellander B.-E. (2014). J. Power Sources.

[cit21] Wang Z., Ouyang D., Chen M., Wang X., Zhang Z., Wang J. (2019). J. Therm. Anal. Calorim..

[cit22] Yuan L., Dubaniewicz T., Zlochower I., Thomas R., Rayyan N. (2020). Process Saf. Environ. Prot..

[cit23] Golubkov A. W., Scheikl S., Planteu R., Voitic G., Wiltsche H., Stangl C., Fauler G., Thaler A., Hacker V. (2015). RSC Adv..

[cit24] Duh Y.-S., Lin K. H., Kao C.-S. (2018). J. Therm. Anal. Calorim..

[cit25] Zhao C., Sun J., Wang Q. (2020). J. Energy Storage.

[cit26] Zalosh R., Gandhi P., Barowy A. (2021). J. Loss Prev. Process. Ind..

[cit27] Md Said M. S., Mohd Tohir M. Z. (2019). Processes.

[cit28] Fang J., Cai J., He X. (2021). Appl. Therm. Eng..

[cit29] Huang P., Ping P., Li K., Chen H., Wang Q., Wen J., Sun J. (2016). Appl. Energy.

[cit30] Gao S., Lu L., Ouyang M., Duan Y., Zhu X., Xu C., Ng B., Kamyab N., White R. E., Coman P. T. (2019). J. Electrochem. Soc..

[cit31] Steen H. , in Handbook of Explosion Prevention and Protection, ed. M. Hattwig and H. Steen, John Wiley & Sons, Weinheim, 2004

[cit32] Shahid S., Agelin-Chaab M. (2022). Energy Convers. Manag..

[cit33] Feng X., Ren D., He X., Ouyang M. (2020). Joule.

[cit34] Vorwerk P., Hahn S.-K., Daniel C., Krause U., Keutel K. (2022). Batteries.

[cit35] Liao Z., Zhang S., Li K., Zhang G., Habetler T. G. (2019). J. Power Sources.

[cit36] Koch S., Birke K. P., Kuhn R. (2018). Batteries.

[cit37] Parekh M. H., Li B., Palanisamy M., Adams T. E., Tomar V., Pol V. G. (2020). ACS Appl. Energy Mater..

[cit38] Cai T., Stefanopoulou A. G., Siegel J. B. (2019). ECS Trans..

[cit39] Bordes A., Marlair G., Zantman A., Chesnaye A., Lore P.-A. L., Lecocq A. (2022). ACS Energy Lett..

[cit40] Roth E. P., Orendorff C. J. (2012). Electrochem Soc. Interface.

[cit41] Barelli L., Bidini G., Ottaviano P. A., Pelosi D., Perla M., Gallorini F., Serangeli M. (2021). J. Energy Storage.

[cit42] Feng X., He X., Ouyang M., Lu L., Wu P., Kulp C., Prasser S. (2015). Appl. Energy.

[cit43] Wilke S., Schweitzer B., Khateeb S., Al-Hallaj S. (2017). J. Power Sources.

[cit44] Hossein Bashiri A., Sangtarash A., Zamani M. (2022). Therm. Sci. Eng. Prog..

[cit45] Ding C., Zhu N., Wang X., Alhadhrami A., Mahmoud M. H. H., Ibrahim M. M., Huang Q., Liu C., Huang M., Wang J. (2022). Adv. Compos. Hybrid Mater..

[cit46] Ding C., Zhu N., Yu J., Li Y., Sun X., Liu C., Huang Q., Wang J. (2022). Case Stud. Therm. Eng..

[cit47] Lyon R. E., Walters R. N. (2016). J. Hazard. Mater..

[cit48] Liu X., Wu Z., Stoliarov S. I., Denlinger M., Masias A., Snyder K. (2016). Fire Saf. J..

[cit49] Ouyang D., Liu J., Chen M., Wang J. (2017). Appl. Sci..

[cit50] Liu X., Stoliarov S. I., Denlinger M., Masias A., Snyder K. (2015). J. Power Sources.

[cit51] Chen M., Zhou D., Chen X., Zhang W., Liu J., Yuen R., Wang J. (2015). J. Therm. Anal. Calorim..

[cit52] Jhu C.-Y., Wang Y.-W., Shu C.-M., Chang J.-C., Wu H.-C. (2011). J. Hazard. Mater..

[cit53] Venkatachalapathy R., Lee C. W., Lu W., Prakash J. (2000). Electrochem. Commun..

[cit54] Chen M., Liu J., Ouyang D., Wang J. (2019). Int. J. Energy Res..

[cit55] Ouyang D., Chen M., Wang J. (2019). J. Therm. Anal. Calorim..

[cit56] Chen M., Dongxu O., Cao S., Liu J., Wang Z., Wang J. (2019). J. Therm. Anal. Calorim..

[cit57] Chen M., Ouyang D., Weng J., Liu J., Wang J. (2019). Process Saf. Environ. Prot..

[cit58] Liu C., Shen W., Liu X., Chen Y., Ding C., Huang Q. (2023). J. Energy Storage.

[cit59] Larsson F., Andersson P., Mellander B.-E. (2016). Batteries.

[cit60] Larsson F., Andersson P., Blomqvist P., Mellander B.-E. (2017). Sci. Rep..

[cit61] Sturk D., Hoffmann L., Ahlberg Tidblad A. (2015). Traffic Inj. Prev..

[cit62] Ribière P., Grugeon S., Morcrette M., Boyanov S., Laruelle S., Marlair G. (2012). Energy Environ. Sci..

[cit63] Fleischhammer M. and Döring H., in Handbuch Lithium-Ionen-Batterien, ed. R. Korthauer, Springer, 2013, 10.1007/978-3-642-30653-2_23

[cit64] Amano K. O. A., Hahn S.-K., Butt N., Vorwerk P., Gimadieva E., Tschirschwitz R., Rappsilber T., Krause U. (2023). Batteries.

[cit65] Zheng S., Wang L., Feng X., He X. (2018). J. Power Sources.

[cit66] Ye J., Chen H., Wang Q., Huang P., Sun J., Lo S. (2016). Appl. Energy.

[cit67] Said A. O., Lee C., Liu X., Wu Z., Stoliarov S. I. (2019). Proc. Combust. Inst..

[cit68] Zhong G., Mao B., Wang C., Jiang L., Xu K., Sun J., Wang Q. (2019). J. Therm. Anal. Calorim..

[cit69] Duh Y.-S., Sun Y., Lin X., Zheng J., Wang M., Wang Y., Lin X., Jiang X., Zheng Z., Zheng S., Yu G. (2021). J. Energy Storage.

[cit70] Essl C., Golubkov A. W., Fuchs A. (2020). J. Electrochem. Soc..

[cit71] Golubkov A. W., Fuchs D., Wagner J., Wiltsche H., Stangl C., Fauler G., Voitic G., Thaler A., Hacker V. (2014). RSC Adv..

[cit72] Lammer M., Königseder A., Hacker V. (2017). RSC Adv..

[cit73] Essl C., Golubkov A. W., Gasser E., Nachtnebel M., Zankel A., Ewert E., Fuchs A. (2020). Batteries.

[cit74] Gully B. , Helgesen H., Skogtvedt J. and Kostopoulos D., Technical Reference for Li-ion Battery Explosion Risk and Fire Suppression, DNV GL, 2019, p. 1025

[cit75] Orendorff C. J. , Lamb J., Steele L. A. M., Spangler S. W. and Langendorf J., Quantification of Lithium-ion Cell Thermal Runaway Energetics, Sandia National Laboratories (SNL-NM), Albuquerque, NM (United States), 2016

[cit76] Diaz F., Wang Y., Weyhe R., Friedrich B. (2019). Waste Manag..

[cit77] Amano K. O. A., Hahn S.-K., Tschirschwitz R., Rappsilber T., Krause U. (2022). Batteries.

[cit78] Rong L. , Cheng X. and Fu Y., 2019 9th International Conference on Fire Science and Fire Protection Engineering, ICFSFPE, 2019, pp. 1–5

[cit79] Lecocq A., Eshetu G. G., Grugeon S., Martin N., Laruelle S., Marlair G. (2016). J. Power Sources.

[cit80] Cai T., Valecha P., Tran V., Engle B., Stefanopoulou A., Siegel J. (2021). eTransportation.

[cit81] Fu Y., Lu S., Li K., Liu C., Cheng X., Zhang H. (2015). J. Power Sources.

[cit82] Lammer M., Königseder A., Gluschitz P., Hacker V. (2018). J. Electrochem. Sci. Eng..

[cit83] Jinghui C. , Fei G., Xiangmei L., Kai Y., Songcen W. and Rongjie Y., Proceedings of the 2017 2nd International Conference on Automation, Mechanical and Electrical Engineering, AMEE 2017, 2017, pp. 199–200, 10.2991/amee-17.2017.40

[cit84] Liu P., Liu C., Yang K., Zhang M., Gao F., Mao B., Li H., Duan Q., Wang Q. (2020). J. Energy Storage.

[cit85] Sturk D., Rosell L., Blomqvist P., Ahlberg Tidblad A. (2019). Batteries.

[cit86] Andersson P., Blomqvist P., Lorén A., Larsson F. (2013). Investigation of fire emissions from Li-ion batteries. SP Report.

[cit87] Nedjalkov A., Meyer J., Köhring M., Doering A., Angelmahr M., Dahle S., Sander A., Fischer A., Schade W. (2016). Batteries.

[cit88] Peng Y., Zhou X., Hu Y., Ju X., Liao B., Yang L. (2020). J. Therm. Anal. Calorim..

[cit89] Wang Z., Zhu K., Hu J., Wang J. (2019). Energy Sci. Eng..

[cit90] Koch S., Fill A., Birke K. P. (2018). J. Power Sources.

[cit91] Wang Q., Huang P., Ping P., Du Y., Li K., Sun J. (2017). J. Loss Prev. Process. Ind..

[cit92] Wang Z., Yang H., Li Y., Wang G., Wang J. (2019). J. Hazard. Mater..

[cit93] Said A. O., Lee C., Stoliarov S. I., Marshall A. W. (2019). Appl. Energy.

[cit94] Peng Y., Yang L., Ju X., Liao B., Ye K., Li L., Cao B., Ni Y. (2020). J. Hazard. Mater..

[cit95] Huang P., Wang Q., Li K., Ping P., Sun J. (2015). Sci. Rep..

[cit96] Jin C., Sun Y., Wang H., Lai X., Wang S., Chen S., Rui X., Zheng Y., Feng X., Wang H., Ouyang M. (2021). J. Power Sources.

[cit97] Golubkov A. W., Planteu R., Krohn P., Rasch B., Brunnsteiner B., Thaler A., Hacker V. (2018). RSC Adv..

[cit98] Huang Z., Liu J., Zhai H., Wang Q. (2021). Energy.

[cit99] Huang L., Zhang Z., Wang Z., Zhang L., Zhu X., Dorrell D. D. (2019). J. Energy Storage.

[cit100] Huang Z., Zhao C., Li H., Peng W., Zhang Z., Wang Q. (2020). Energy.

[cit101] Zhu N., Wang X., Huang Q., Ding C., Wang J. (2023). J. Therm. Anal. Calorim..

[cit102] Huang Z., Li X., Wang Q., Duan Q., Li Y., Li L., Wang Q. (2021). Int. J. Heat Mass Transfer.

[cit103] Feng X., Sun J., Ouyang M., Wang F., He X., Lu L., Peng H. (2015). J. Power Sources.

[cit104] Chen M., He Y., De Zhou C., Richard Y., Wang J. (2016). Fire Technol..

[cit105] Li H., Duan Q., Zhao C., Huang Z., Wang Q. (2019). J. Hazard. Mater..

[cit106] Ditch B. , Development of Protection Recommendations for Li-ion Battery Bulk Storage Sprinklered Fire, Report, FM Global, 2016

[cit107] Ditch B. and de Vries J., Fammability Characterization of Lithium ion Batteries in Bulk Storages, Report, FM Global, 2013

[cit108] Maloney T. , Lithium battery thermal runaway vent gas analysis, Report, US Department of Transportation, 2016

[cit109] Un C., Aydin K. (2021). Vehicles.

[cit110] Said A. O., Garber A., Peng Y., Stoliarov S. I. (2022). Fire Technol..

[cit111] Sun H., Zhang L., Duan Q. L., Wang S., Sun S., Sun J. H., Wang Q. S. (2022). Process Saf. Environ. Prot..

[cit112] Liu X., Xu D., Meng X., Lu Z., Chen Y., Liu C., Huang Q. (2022). Case Stud. Therm. Eng..

[cit113] Held M., Tuchschmid M., Zennegg M., Figi R., Schreiner C., Mellert L. D., Welte U., Kompatscher M., Hermann M., Nachef L. (2022). Renew. Sustain. Energy Rev..

[cit114] Lecocq A. , Bertana M., Truchot B. and Marlair G., Conference proceedings of Fires in vehicles, 2012, pp. 183–193

[cit115] Willstrand O. , Bisschop R., Blomqvist P., Temple A. and Anderson J., Toxic Gases from Fire in Electric Vehicles, 2020

[cit116] Liu P., Li Y., Mao B., Chen M., Huang Z., Wang Q. (2021). Appl. Therm. Eng..

[cit117] Mao B., Liu C., Yang K., Li S., Liu P., Zhang M., Meng X., Gao F., Duan Q., Wang Q., Sun J. (2021). Renew. Sustain. Energy Rev..

[cit118] Cheng X., Li T., Ruan X., Wang Z. (2019). Energies.

[cit119] Long R. T. and Blum A., Hazard Assessment of Lithium Ion Battery Energy Storage Systems, NFPA, 2016

[cit120] TC 65/SC 65B International Electrotechnical Commission, (2013). IEC 60584-1: Thermocouples—Part 1: EMF Specifications and Tolerances.

[cit121] Rappsilber T., Krüger S. (2018). Fire Saf. J..

[cit122] European Committee for Standardization, CEN/TC 127/WG 4, DIN EN 13823:2020-09 — Reaction to fire tests for building products - Building products excluding floorings exposed to the thermal attack by a single burning item, 2020, 10.31030/3115730

[cit123] Ping P., Kong D., Zhang J., Wen R., Wen J. (2018). J. Power Sources.

[cit124] Coman P. T., Rayman S., White R. E. (2016). J. Power Sources.

[cit125] Rappsilber T., Yusfi N., Krüger S., Hahn S.-K., Fellinger T.-P., Krug von Nidda J., Tschirschwitz R. (2023). J. Energy Storage.

[cit126] National Research Council Subcommittee on Acute Exposure Guideline Levels , in Acute Exposure Guideline Levels for Selected Airborne Chemicals: Volume 4, National Academies Press (US), Washington (DC), 2004, 10.17226/10902 25032305

[cit127] Ludwig H. R. , Cairelli S. G. and Whalen J. J., Documentation for immediately dangerous to life or health concentrations, IDLHS, 1994

[cit128] Brandes E. , Dietlen S., Hieronymus H., Krause U., Plewinsky B., Redeker T., Schröder V. and Hoyermann K., in Handbook of Explosion Prevention and Protection, ed. M. Hattwig and H. Steen, John Wiley & Sons, Weinheim, 2004

[cit129] Hill D. , Warner N. and Kovacs III W., Considerations for ESS fire safety, Report, DNV GL, Oslo, Norway, 2017

[cit130] National Research Council Subcommittee on Acute Exposure Guideline Levels , in Acute Exposure Guideline Levels for Selected Airborne Chemicals: Volume 8, National Academies Press, US, 2009, 10.17226/12770

